# Phylogenetic and transcriptomic characterization of insulin and growth factor receptor tyrosine kinases in crustaceans

**DOI:** 10.3389/fendo.2024.1379231

**Published:** 2024-04-04

**Authors:** Kaylie A. Flores, Jorge L. Pérez-Moreno, David S. Durica, Donald L. Mykles

**Affiliations:** ^1^ Department of Biology, Colorado State University, Fort Collins, CO, United States; ^2^ Department of Biology, University of Oklahoma, Norman, OK, United States; ^3^ Bodega Marine Laboratory, University of California, Davis, Bodega Bay, CA, United States

**Keywords:** receptor tyrosine kinase (RTK), epidermal growth factor receptor (EGFR), insulin receptor (INSR), fibroblast growth factor receptor (FGFR), vascular endothelial growth factor receptor (VEGF), platelet-derived growth factor receptor (PDGFR), crustacea, CrusTome

## Abstract

Receptor tyrosine kinases (RTKs) mediate the actions of growth factors in metazoans. In decapod crustaceans, RTKs are implicated in various physiological processes, such molting and growth, limb regeneration, reproduction and sexual differentiation, and innate immunity. RTKs are organized into two main types: insulin receptors (InsRs) and growth factor receptors, which include epidermal growth factor receptor (EGFR), fibroblast growth factor receptor (FGFR), vascular endothelial growth factor receptor (VEGFR), and platelet-derived growth factor receptor (PDGFR). The identities of crustacean RTK genes are incomplete. A phylogenetic analysis of the CrusTome transcriptome database, which included all major crustacean taxa, showed that RTK sequences segregated into receptor clades representing InsR (72 sequences), EGFR (228 sequences), FGFR (129 sequences), and PDGFR/VEGFR (PVR; 235 sequences). These four receptor families were distinguished by the domain organization of the extracellular N-terminal region and motif sequences in the protein kinase catalytic domain in the C-terminus or the ligand-binding domain in the N-terminus. EGFR1 formed a single monophyletic group, while the other RTK sequences were divided into subclades, designated InsR1-3, FGFR1-3, and PVR1-2. In decapods, isoforms within the RTK subclades were common. InsRs were characterized by leucine-rich repeat, furin-like cysteine-rich, and fibronectin type 3 domains in the N-terminus. EGFRs had leucine-rich repeat, furin-like cysteine-rich, and growth factor IV domains. N-terminal regions of FGFR1 had one to three immunoglobulin-like domains, whereas FGFR2 had a cadherin tandem repeat domain. PVRs had between two and five immunoglobulin-like domains. A classification nomenclature of the four RTK classes, based on phylogenetic analysis and multiple sequence alignments, is proposed.

## Introduction

Receptor tyrosine kinases (RTKs) are cell membrane receptors that mediate the actions of peptide growth factors in metazoan organisms. In humans, there are 55 RTKs organized into 19 subfamilies or classes, as it is now recognized that three kinases in the lemur class phosphorylate serine/threonine residues (1, 2). Of these, five RTK classes are the most common across metazoan taxa: epidermal growth factor receptor (EGFR; Class I); insulin receptor, IGF1 receptor, and the insulin receptor-related receptor (InsR; Class II); platelet-derived growth factor receptor (PDGFR; Class III); vascular endothelial growth factor receptor (VEGFR; Class IV); and fibroblast growth factor receptor (FGFR; Class V) (3, 4). All RTKs share a similar organization with an N-terminal extracellular region containing dimerization and ligand-binding domains, an α-helical transmembrane domain, and a C-terminal region containing a tyrosine kinase domain and tyrosine-rich C-terminus. An intracellular juxtamembrane segment, located between the transmembrane and tyrosine kinase domains, mediates autoinhibition by interacting with the activation loop in the kinase domain (3–6). InsR is a heterotetramer of disulfide-linked αβ subunits resulting from furin cleavage of a protein precursor (7). InsR ligands include insulin, insulin-like growth factors (IGFs), and insulin-like peptides (ILPs) (3, 7). By contrast, EGFRs, FGFRs, PDGFRs, and VEGFRs are monomers in the cell membrane and form homodimers or heterodimers upon binding of their respective ligands and activation (3, 4, 8). Isoforms that differ in ligand binding affinity and specificity are common (3, 7, 9–11). Receptor activation results in autophosphorylation of tyrosines in the juxtamembrane segment and the C-terminus and phosphorylation of signal transduction proteins that are recruited to the receptor (3–7). RTKs can activate various signal transduction pathways, such as MAPK-ERK, PI3K/Akt/mTOR, JAK/STAT, and PLC/PKC, that stimulate cell proliferation, growth, and metabolism (1, 3, 5–7, 9–12).

RTK classes are distinguished by the functional domains in the N-terminal extracellular region (1, 6). InsRs are characterized by two leucine-rich repeats (Receptor L1 and L2) flanking a furin-like cysteine-rich domain and two fibronectin type 3 (FN3) domains in the α subunit (7, 13). EGFRs are characterized by L1 and L2 domains alternating with two furin-like cysteine-rich domains (6, 8, 14). FGFRs have three immunoglobulin-like domains (D1, D2, and D3), with a seven or eight amino acid “acid box” linking D1 and D2 (6, 9). PDGFRs and VEGFRs are structurally related, which suggests a common origin. PDGFR and VEGFR have five or seven immunoglobulin-like domains (D1 to D7), respectively, that are involved in ligand binding (6, 11, 15).

In crustaceans, RTKs have been implicated in diverse physiological processes, particularly those involving reproduction, development, immunity, and growth. EGFR plays a role in ovarian development in the mud crab, *Scylla paramamosain* (16). EGFR and FGFR are linked to the ability of *S. paramamosain* and red swamp crayfish (*Procambarus clarkii*) to mount immune responses to pathogens (17–19). Knockdown of *Mr-EGFR* slows organismal growth, but it has no effect on molting frequency in freshwater prawn, *Macrobrachium rosenbergii* (20). By contrast, knockdown of *Mr-InsR* has no effect on organismal growth, but results in abnormalities in development of male sex characteristics and reproductive organs (21, 22). In Chinese mitten crab, *Eriocheir sinensis*, *Es-InsR* expression is increased in limb regenerates and blocking InsR signaling with GSK1838705A slows regenerate growth (23). A male-specific InsR may be involved in sexual differentiation in Pacific whiteleg shrimp, *Litopenaeus vannamei*; Chinese shrimp, *Fenneropenaeus chinensis*; Eastern spiny lobster, *Sagmariasus verreauxi*; and *S. paramamosain* (24–27). VEGFR/PDGFR signaling is involved in immune responses to viral infection in *L. vannamei*; in hemopoiesis in signal crayfish, *Pascifasticus leniusculus*; and in regulating lipid metabolism in *S. paramamosain* (28–30).

Transcriptomics has assisted in the identification of RTKs in crustacean tissues, but these receptors have not been fully characterized (17, 26, 31–39). Moreover, annotation and characterization of RTKs in diverse crustacean taxa has been hampered by databases that are limited to a relatively small number of species and taxonomic groups. Consequently, the number of RTK genes and/or isoforms present in crustaceans is unknown. Additionally, the patterns of evolution and diversification of RTKs across the Pancrustacea remain to be elucidated (31). CrusTome, a comprehensive multi-species database of crustacean transcriptomes (40), was used to identify contiguous sequences encoding insulin, EGF, FGF, and PDGF/VEGF (PV) receptors in malacostracan and non-malacostracan crustaceans. A similar approach was used to identify G protein-coupled receptor candidates for crustacean hyperglycemic hormone neuropeptides (41). Characterization of decapod RTKs was emphasized, particularly in blackback land crab, *Gecarcinus lateralis* and green shore crab, *Carcinus maenas*, which have served as models for molting physiology for decades (42–52). Moreover, *C. maenas* is an invasive species that has established populations in temperate coastal regions (53). Its rapid growth and tolerance of a wide range of environmental conditions have contributed to its success (54–56). In *G. lateralis*, Gl-InsR, Gl-EGFR, Gl-FGFR, and other RTK signaling genes are expressed in transcriptomes of the molting gland (Y-organ), suggesting that growth factors have a direct effect on the synthesis of steroid molting hormones (ecdysteroids) (32, 33, 38, 46). RTKs in *C. maenas* have not been characterized. Phylogenetic analysis and multiple sequence alignments revealed a rich diversity of RTK genes and isoforms. A classification nomenclature, based on InsR, EGFR, FGFR, and PVR clades and subclades, is proposed.

## Materials and methods

Protein reference sequences for each receptor were collected from the NCBI GenBank database with a focus on arthropod sequences when available (**Supplementary Material 1**). Four iterative BLAST searches using these reference sequences against the CrusTome database (v.0.1.0) were then conducted to ensure that all possible matching sequences were found for a comprehensive phylogenetic analysis (40, 57). Using Multiple Alignment using Fast Fourier Transform (MAFFT; v.7.490; (58), the BLAST search hits and the original reference sequence dataset were aligned with settings optimized for multi-domain proteins (as per (59) and to place a higher importance on accuracy rather than speed (*-dash-originalseqonly -genfpair -maxiterate 1000*). The *-dash* parameter allowed MAFFT to refine the alignment by employing sequences from the Database of Aligned Structural Homologs (DASH; (60), which includes structural information to improve the alignment processes. Subsequently, ClipKIT (61), with the smart-gap parameter, was used to trim the alignment gaps while retaining phylogenetically informative sites for the most accurate phylogenetic inference. A maximum-likelihood phylogenetic reconstruction was undertaken with IQ-TREE (62) to accurately create a phylogeny of the sequences found for each given receptor using the models of evolution indicated by ModelFinder (63); VT+R8 for InsR, JTT+I+I+R6 for EGFR, VT+F+R7 for FGFR, and WAG+F+I+I+R7 for PDGFR/VEGFR). These trees were refined to reduce partial sequences (less than 200 aa for EGFR, 350 aa for InsR, 200 aa for PDGFR/VEGFR, 170 aa for FGFR), sequences with ambiguous or unknown residues (often found in *Daphnia* predicted transcriptomes), and any sequences that confidently lacked the domain organization of RTKs. Final phylogenies were reconstructed using the pruned input dataset. All final trees, their corresponding input files, and the alignments for *G. lateralis* and *C. maenas* can be found in **Supplementary Material 2**. Branch support for the finalized phylogenetic reconstructions was assessed via two complementary methods, the Ultra-Fast Bootstrap approximation (UFBoot; 1,000 iterations) and an approximate Bayes test (64–66).

A multiple sequence alignment restricted to brachyuran species was performed following the MAFFT strategy outlined above, to identify putative residues of structural and/or functional significance conserved across taxa (**Supplementary Material 3**). This alignment was subsequently used as input for the Motifs from Annotated Groups in Alignments (MAGA) tool (67) to identify motifs that could be employed to discriminate between RTK classes without the need of large-scale phylogenetic analyses. This tool consisted of a supervised method to detect motifs that can identify sites of structural, functional, and/or evolutionary significance based on sequence conservation within and across groups, as defined by the previous phylogenetic analyses. Multiple sequence alignments were produced to assess sequence content and conservation across receptor types and subclades among select decapod species. These alignments were generated with the MAFFT strategy and subsequently visualized with a custom script (code available at https://github.com/invertome/scripts/tree/main/plots; from (41). Additionally, the script generated sequence logo plots depicting the proportion of each residue found per alignment site. Amino acid residue colors that are proximal in color space, in both the alignments and logo plots, denote similarities in physicochemical characteristics of the corresponding residues (68). Additionally, further examination using NCBI’s Conserved Domain Database (69) assisted with the comparison and identification of sequences. In this study, the protein sequences were analyzed using a database of recognized domains, which revealed commonly-found domains in RTKs, as well as domains that suggested a non-RTK identity.

## Results

### Phylogenetic analysis of receptor tyrosine kinases

**Figure 1:**
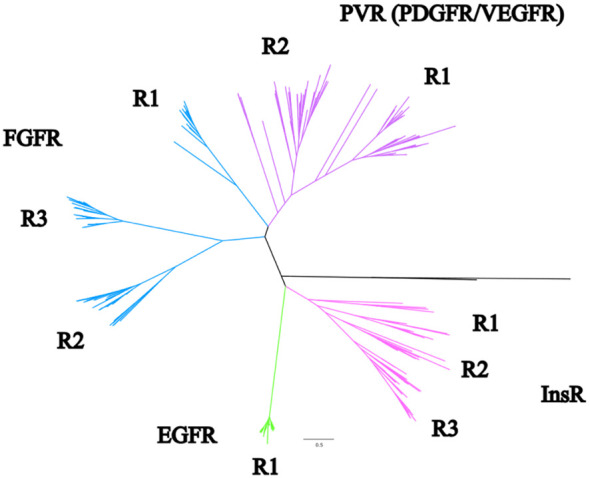
Phylogenetic analysis of pancrustacean receptor tyrosine kinases. A phylogenetic tree synthesized using all the initial references sequences, all decapod sequences, and the identified sequences found in other species from other studies using a WAG+F+R9 substitution model of evolution. The pink clade represents an overall InsR identity. The green clade is EGFR, and the blue and purple are FGFR and PVR, respectively. Non-RTK and non-decapod sequences were removed for clarity. The full unedited tree can be found in **Supplementary Material 2**.

Maximum-likelihood phylogenetic analysis of crustacean RTKs in the CrusTome database produced a well-supported tree with four major clades, corresponding to InsR, EGFR, FGFR, and PVR classes (**Figure 1**). The EGFR class consisted of a single monophyletic group, designated EGFR1 (**Figure 1**). The other classes segregated into subclades denoting possible receptor subtypes. The analyses supported a classification nomenclature based on these clades and subclades. The InsR clade had three subclades, designated InsR1, InsR2, and InsR3; the FGFR clade had three subclades, designated FGFR1, FGFR2, and FGFR3; and the PVR clade had two subclades, designated PVR1 and PVR2 (**Figure 1**; full unedited tree provided in **Supplementary Material 2**).

**Table 1** summarizes the distribution of RTK sequences obtained from pancrustacean and tardigrade transcriptomes in the CrusTome database. The 51 decapod species had the highest number of RTK sequences, which included 60 InsR sequences (**Table 1**). Fewer InsR sequences were identified in non-decapod taxa; the next highest number was eight sequences in 22 isopod species, followed by four sequences in two euphausiid species (**Table 1**). InsR sequences were not obtained from transcriptomes from the other 11 taxa (**Table 1**). By contrast, growth factor receptor sequences were well represented in seven pancrustacean taxa: a total of 344 in decapods; 128 in 22 isopod species; 97 in 26 amphipod species; 95 in two branchiopod species; 55 in two euphausiid species; 53 in eight copepod species; and 33 in three hexapod species (**Table 1**).

**Table 1:** Summary of CrusTome pancrustacean and tardigrade receptor tyrosine kinase sequences.

Taxon	# of Species	InsR	EGFR	FGFR	PVR	Total
Decapoda	51	60	77	129	138	404
Amphipoda	26	0	34	59	4	97
Isopoda	22	8	53	62	13	136
Copepoda	8	0	9	38	6	53
Euphausiacea	2	4	26	24	5	59
Branchiopoda	2	0	19	15	61	95
Bathynellacea	1	0	2	3	1	6
Cirripedia	1	0	4	0	4	8
Remipedia	2	0	2	3	0	5
Stomatopoda	1	0	2	0	3	5
Leptostraca	1	0	0	1	0	1
Mysida	1	0	0	1	0	1
Hexapoda	3	0	5	11	17	33
Tardigrada	2	0	3	4	0	7

Taxonomic distribution of insulin receptor (InsR), epidermal growth factor receptor (EGFR), fibroblast growth factor receptor (FGFR), and platelet-derived growth factor/vascular endothelial growth factor receptor (PVR) sequences identified in the CrusTome 1.0 database and included in the final phylogenies (40). RTK sequences previously deposited in Genbank are not included. Sequences available in **Supplementary Material 1**.

### Crustacean insulin receptors

**Figure 2:**
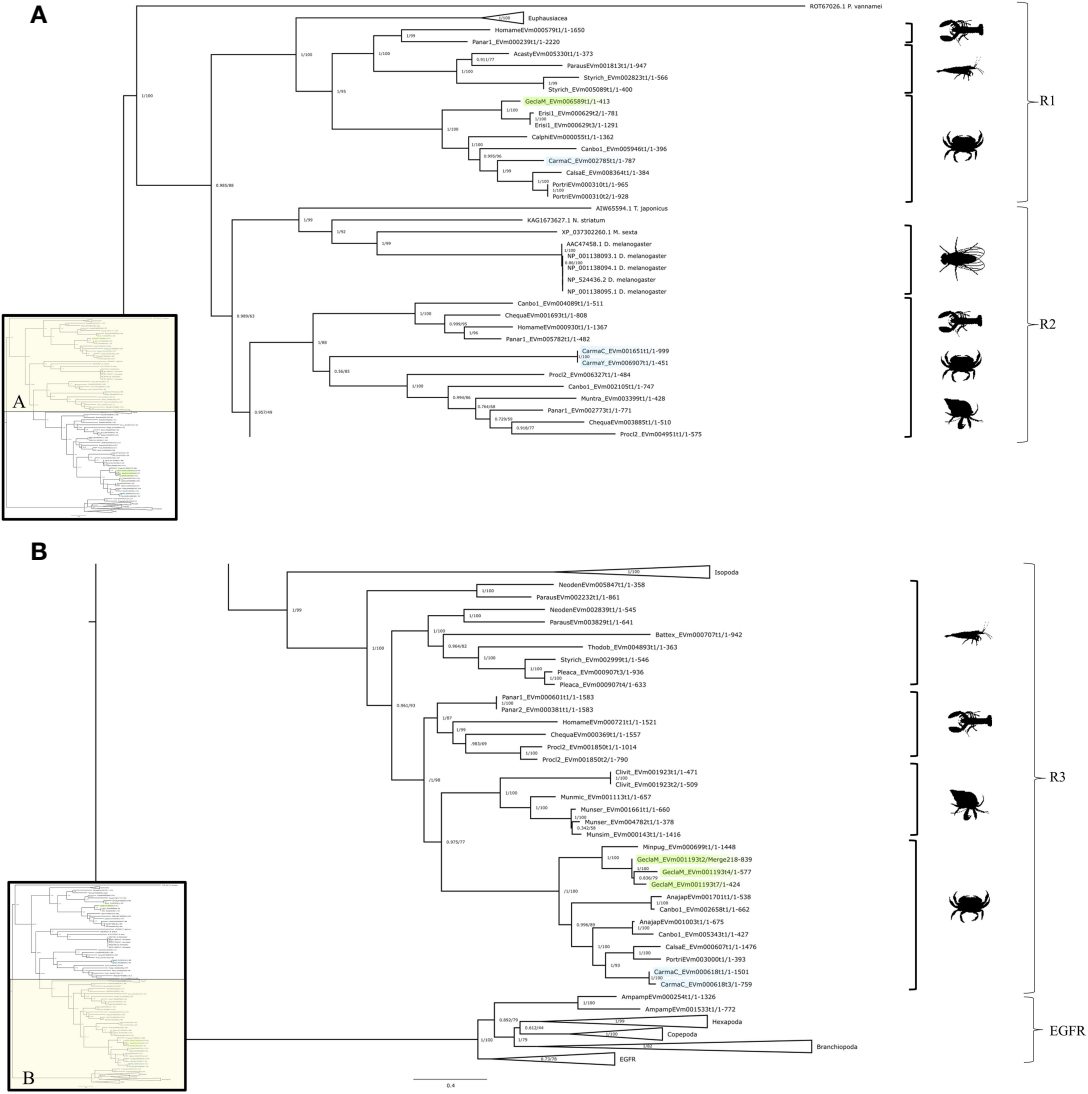
Insulin receptor phylogeny. A phylogenetic tree of InsR exhibiting an array of species, including two *G. lateralis* genes and three *C. maenas* genes. Inset depicts entire tree, divided into sections **(A)** (InsR1 and InsR2) and **(B)** (InsR3 and EGFR), for orientation. Further analysis of the conserved domains of the ROT67026.1 sequence suggested an identity other than an RTK, making it an outgroup for the tree. Support values correspond to the approximate Bayes test and the Ultra-Fast Bootstrap approximation with the VT+R8 substitution model of evolution. Images from PhyloPic: *Homarus* (lobster) by Steven Traver; *Caridina multidentate* (shrimp) by Douglas Teles da Rosa; *Metacarcinus magister* (Dungeness crab) by Harold Eyster; *Pagurus pubescens* (hermit crab) by T. Michael Keesey; and *Sophophora melanogaster* (fly) by Andy Wilson.

The InsR tree had four subclades that reflected crustacean taxonomic classifications with high bootstrap values supporting each branch (**Figure 2**). There were three InsR subclades, designated InsR1, InsR2, and InsR3 (**Figure 2**). A conserved domain search identified the fourth subclade as EGFR, as the sequences contained a PTKc/EGFR-like catalytic domain (**Figure 2B**; see section “Crustacean epidermal growth factor receptors” below). The InsR1 subclade included sequences from Hexapoda and Malacostraca, including Euphausiacea (krill) and Decapoda (Achelata, Astacidea, Brachyura, and Caridea) (**Figure 2A**). The InsR2 subclade included sequences from Hexapoda and Decapoda (Achelata, Anomura, Astacidae, and Brachyura) (**Figure 2A**). The InsR3 subclade included sequences from Isopoda and Decapoda (Achelata, Anomura, Astacidea, Caridea, and Brachyura) (**Figure 2B**).

**Figure 3:**
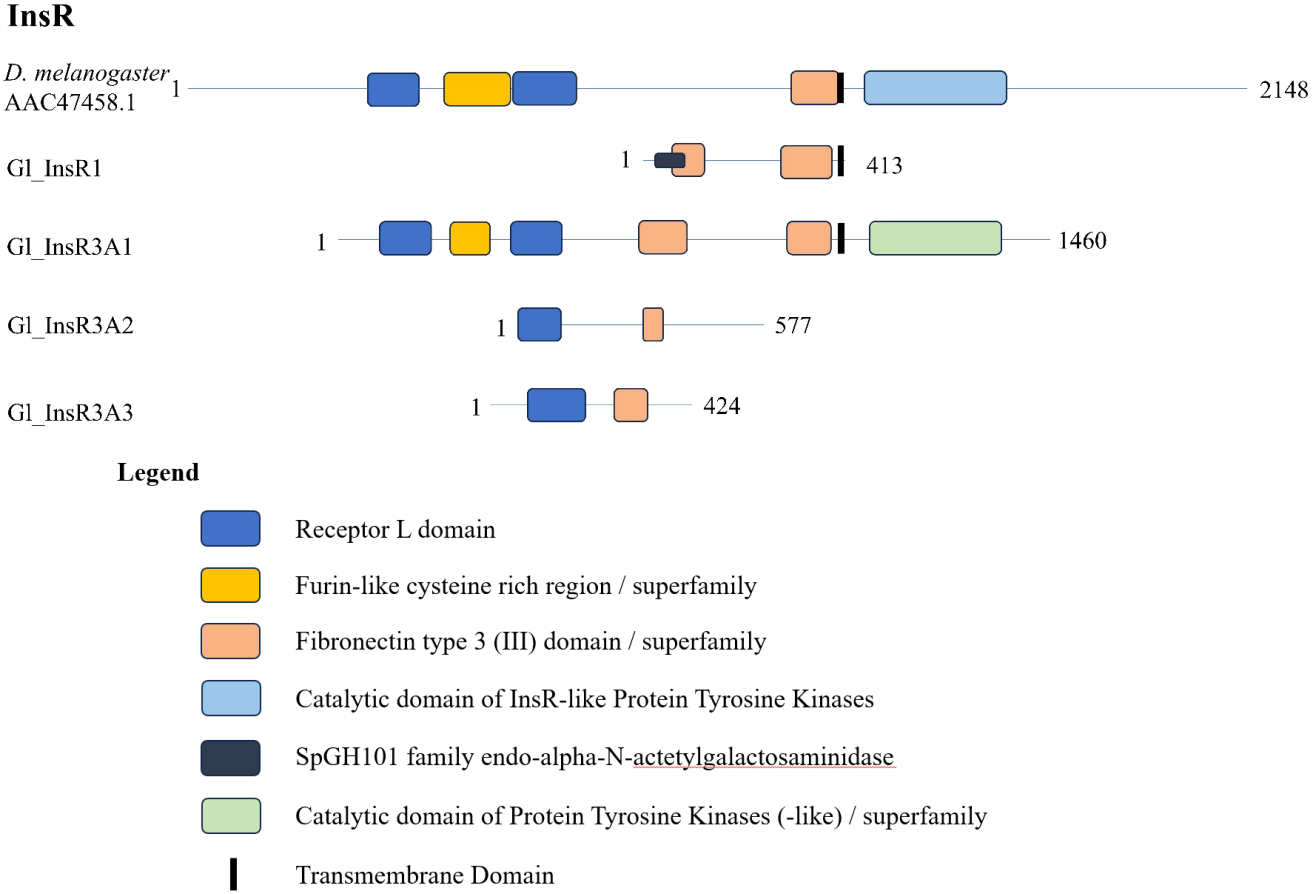
Domain organization of *Drosophila* and decapod insulin receptors. Listed sequences include a model organism (*D. melanogaster*), *G. lateralis* sequences, and identified genes in other species using the classification as listed in the original referenced studies (**Table 2**).

The contig sequences encoding InsRs from selected decapod species are presented in **Table 2**. The InsR1 subclade was represented by single contigs in *G. lateralis* (*Gl-InsR1*), *C. maenas* (*Cm-InsR1*), *Cancer borealis* (*Cb-InsR1*), and *Eriochier sinensis* (*Es-InsR1*) (**Table 2**). *Gl-InsR1* was a partial sequence, as it lacked the kinase domain (**Figure 3**). Its identity was confirmed by its phylogenetic proximity to Es-InsR1 (Erisi1_EVm000629t3), which had all the domains identified in RTKs, including the protein tyrosine kinase domain (**Figure 2A**). The *Gl-InsR1* contig sequence had a transmembrane domain and two fibronectin type 3 (FN3) domains in the N-terminal region (**Figure 3**). A *Gl-InsR2* contig sequence was not extracted from the CrusTome database. However, *InsR2* contigs were identified in *C. maenas*, *C. borealis*, *S. paramamosain*, *F. chinensis*, and *S. verreauxi* (**Table 2**). In the third subclade, three *Gl-InsR3* isoforms, designated *Gl-InsR3-A1*, *Gl-InsR3-A2*, and *Gl-InsR3-A3*, were identified (**Figure 2B**, **Table 2**). A full-length sequence of *Gl-InsR3-A1* was obtained manually by combining three overlapping partial contig sequences (GeclaM_EVm001193t2/2, GeclaM_EVm001193t2/1, and GeclaM_EVm001193t2/8). *Gl-InsR3-A2* and *Gl-InsR3-A3* were partial sequences (**Figure 3**). DNA alignment identified highly conserved regions shared between the *Gl-InsR3-A1*, *-A2*, and *-A3* sequences (**Supplementary Material 2**). Gl-InsR3-A1 contained two leucine-rich repeat (Receptor L1 and L2) domains, a furin-like cysteine-rich region, two FN3 domains, a transmembrane domain, and a protein tyrosine kinase catalytic domain (**Figure 3**). Two *C. maenas* isoforms, designated *Cm-InsR3-A1* and *Cm-InsR3-A2*, were also found in this subclade (**Table 2**, **Figure 2B**).

**Table 2:** Classification of decapod insulin receptors.

Name	Species	Tissue	Transcript ID	Accession #
*Gl-InsR1*	*G. lateralis*	YO	GeclaM_Evm006589t1*	OR767207
*Cm-InsR1*	*C. maenas*	CNS	CarmaC_Evm002785t1	OR767208
*Es-InsR1*	*E. sinensis*	MD	Erisi1_Evm000629t2* Erisi1_Evm000629t3	
*Cb-InsR1*	*C. borealis*	N	Canbo1_Evm005946t1*	
*Cm-InsR2*	*C. maenas*	CNS YO	CarmaC_Evm001651t1 CarmaY_Evm006907t1*	OR767210 OR767209
*Cb-InsR2*	*C. borealis*	N	Canbo1_Evm004089t1* Canbo1_Evm002105t1	
*Sp-InsR2*	*S. paramamosain*	Testis	Sp-IR^1^	OQ361826
*Fc-InsR2*	*F. chinensis*	Testis, AG	Fc-IAGR^2^	AVU05021.1
*Lv-InsR2*	*L. vannamei*	Unknown	Lv-IR^3^	XP027207730.1
*Sv-InsR2*	*S. verreauxi*	Various	Sv-TKIR^4^	ANC28181.1
*Gl-InsR3-A1*	*G. lateralis*	YO	GeclaM_Evm001193t2†	OR772928
*Gl-InsR3-A2*	*G. lateralis*	YO	GeclaM_Evm001193t4*	OR772876
*Gl-InsR3-A3*	*G. lateralis*	YO	GeclaM_Evm001193t7*	OR772877
*Cm-InsR3-A1*	*C. maenas*	CNS	CarmaC_Evm00618t1	OR772927
*Cm-InsR3-A2*	*C. maenas*	CNS	CarmaC_Evm00618t3	OR772929
*Mr-InsR3*	*M. rosenbergii*	Unknown	Mr-IR^5^	AKF17681.1
*Es-InsR3*	*E. sinensis*	Unknown	Es-InR^6^	XP050738123.1
*Cb-InsR3*	*C. borealis*	N	Canbo1_Evm002658t1 Canbo1_Evm005343t1	

Contigs encoding InsRs in the CrusTome 1.0 database and previously identified InsRs in other decapods. Gene names are the proposed classification, based on clades and subclades from taxonomically comprehensive phylogenetic analyses. Both Cm-InsR2 sequences have the same classifications, as one was a partial sequence of the other a full-length sequence. Species: *Gecarcinus lateralis, Carcinus maenas, Cancer borealis, Sagmariasus verreauxi, Fenneropenaeus chinensis, Litopenaeus vannamei, Scylla paramamosain, Macrobrachium rosenbergii*, and *Eriocheir sinensis*. Tissue sources: AG, androgenic gland; CNS, central nervous system; MD, multiple developmental stages of whole larvae; and N, neural tissues. GenBank accession numbers included, if known. Sequences are available in **Supplementary Material 1**. Asterisk (*) indicates partial sequence; open reading frame incomplete. †Combination of three partial contigs: GeclaM_Evm001193t2/2, GeclaM_Evm001193t2/1, and GeclaM_Evm001193t2/8.

^1^from (27).

^2^from (25).

^3^from (24).

^4^from (26).

^5^from (21, 27).

^6^from (23).

**Figure 4:**
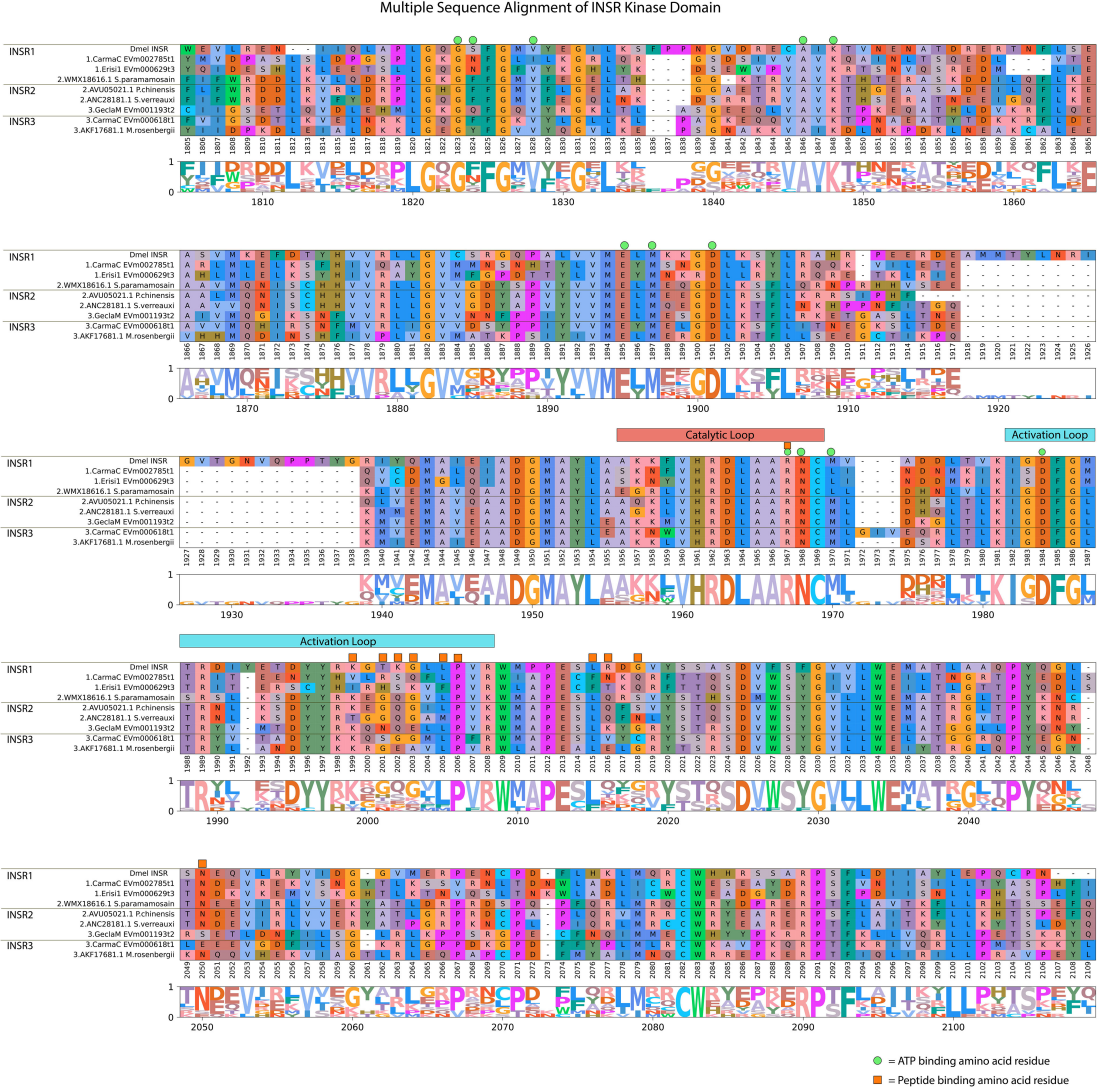
Multiple sequence alignment and logo plot of the catalytic domain of *Drosophila* and decapod insulin receptors. Includes representative species from **Table 2** (*Gecarcinus lateralis*, *Carcinus maenas, Cancer borealis, Sagmariasus verreauxi, Fenneropenaeus chinensis, Scylla paramamosain, Macrobrachium rosenbergii*, and *Eriocheir sinensis*) and *Drosophila melanogaster* INSR1 (Accession: AAC47458.1) as a reference for comparison. The alignment illustrates the composition and length of conserved regions within subclades that reflect putative differences in ligands and/or binding affinities between receptor types. Catalytic loop and activation loop regions are demarcated by red and blue rectangles, respectively. ATP-binding and peptide-binding amino acid residues are annotated with green circles and orange squares, respectively, above the reference position. Partial sequences were excluded for ease of visualization and interpretation. MSA color scheme corresponds to similarities in physicochemical properties of amino acid residues. Logo plot illustrates conserved amino acid residues as a proportion of all the sequences included.

Multiple sequence alignment of the identified decapod InsR sequences kinase domain with a *Drosophila melanogaster* reference revealed remarkable conservation of ATP-binding sites (10 out of 12 sites), including those outside (**Figure 4**; reference alignment positions #1823, #1846, #1848, #1895, #1897, and #1901) and in the loop regions (reference alignment positions #1967, #1968, #1970, and #1984). Peptide-binding residues on the other hand were only conserved across InsR subtypes in 5 out of 11 identified sites (**Figure 4**, reference alignment positions #1967, #1968, and #2006; positions #2015, #2050 in the loop region). MAGA search identified conserved motifs in decapod InsR proteins (67). A VHRDLAARNC motif, located in the catalytic loop, was conserved in all decapod InsRs, which distinguished the InsRs from the decapod growth factor RTKs (**Table 3**, **Figure 4**, reference alignment positions #1960 to #1969; **Supplementary Material 1**). The three InsR subclades were distinguished by motif sequences in a 20-amino acid stretch located proximal to the beginning of the first FN3 domain in the N-terminus. There were four residues in the motif that were conserved in all decapod InsRs (**Table 3**, **Supplementary Material 4**). The 20-amino acid sequence was highly conserved in InsR1 (**Table 3**, **Supplementary Material 4**, reference sequence positions #840 to #859). Although the motif sequences varied among InsR2 and InsR3 subclades, there were consistent differences in the sequences to distinguish the two subclades (**Table 3**).

**Table 3:** Motif sequences distinguishing decapod insulin receptors.

Receptor	FN3 sequences	Catalytic loop sequence
InsR1	R**YA**VY**V**ETDTVADADIGAR**S**	**VHRDLAARNC**
InsR2	R**YA**Vx**V**KxxSLxSSxxGAQ**S**	**VHRDLAARNC**
InsR3	x**YA**xY**V**xxYYTDxxKxxSR**S**	**VHRDLAARNC**

InsR1, InsR2, and InsR3 were distinguished by a 20-amino acid motif sequence located near the N-terminal end of the first FN3 domain in the N-terminal region. All InsRs had a conserved 10-amino acid sequence in the catalytic loop in the catalytic domain in the C-terminal region. Residues that are identical between all the sequences from **Table 2** are indicated by bold font. Consensus sequences were obtained using the MAGA tool (67) and multiple sequence alignment (**Figure 4**).

### Crustacean epidermal growth factor receptors

**Figure 5:**
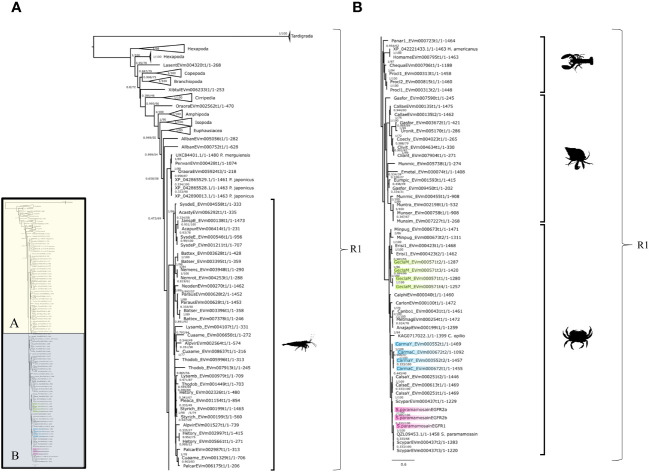
Epidermal growth factor receptor phylogeny. A phylogenetic tree of EGFR containing four *G. lateralis* and four *C. maenas* isoforms. Inset depicts entire tree, divided into sections **(A)** and **(B)**, for orientation. Support values correspond to the approximate Bayes test and the Ultra-Fast Bootstrap approximation with the JTT+I+I+R6 substitution model of evolution. Images from PhyloPic, as credited in **Figure 2**, represent Decapod-only subclades.

Phylogenetic analysis showed that the crustacean EGFRs grouped as a single clade, designated EGFR1, with remarkable conservation across all pancrustacean taxa, including Branchiopoda, Cirripedia, Copepoda, Decapoda, and Hexapoda (**Figures 5A, B**). Within the decapods, EGFR1 sequences clustered into discrete taxonomic groups (Achelata, Anomura, Astacidea, Brachyura, and Caridea) (**Figure 5**). Multiple EGFR1 isoforms were identified in *G. lateralis*, *C. maenas*, and *S. paramamosain* (**Table 4**). DNA alignment of the *G. lateralis* isoforms showed that the four sequences, designated *Gl-EGFR1-A1*, *-A2*, *-A3*, and *-A4*, were likely products of a single gene (**Figure 5B**, **Table 4**, **Supplementary Material 2**). Four *C. maenas* isoforms, designated *Cm-EGFR1-A1, -A2*, *-A3*, and *-A4*, grouped proximally to *G. lateralis* and other brachyurans (**Figure 5B**, **Table 4**, **Supplementary Material 2**). In *S. paramamosain*, three contig sequences obtained from the CrusTome database grouped with three previously-described EGFR1 coding sequences (17) (**Figure 5B**, **Table 4**, **Supplementary Material 2**).

**Table 4:** Classification of decapod EGF receptors.

Name	Species	Tissue	Transcript ID	Accession #
*Gl-EGFR1-A1*	*G. lateralis*	YO	GeclaM_Evm000571t2*	OR772878
*Gl-EGFR1-A2*	*G. lateralis*	YO	GeclaM_Evm000571t3	OR772879
*Gl-EGFR1-A3*	*G. lateralis*	YO	GeclaM_Evm000571t1*	OR772880
*Gl-EGFR1-A4*	*G. lateralis*	YO	GeclaM_Evm000571t4	OR772881
*Cm-EGFR1-A1*	*C. maenas*	YO	CarmaY_Evm000552t1	OR772882
*Cm-EGFR1-A2*	*C. maenas*	CNS	CarmaC_Evm000672t2	OR772883
*Cm-EGFR1-A3*	*C. maenas*	YO	CarmaY_Evm000552t2	OR772884
*Cm-EGFR-1A4*	*C. maenas*	CNS	CarmaC_Evm000672t1	OR772885
*Lv-EGFR1*	*L. vannamei*	PW	PenvanEVm000428t1	
*Es-EGFR1*	*E. sinensis*	MD	Erisi1_Evm000423t1 Erisi1_Evm000423t2	
*Cb-EGFR1*	*C. borealis*	N	Canbo1_Evm000431t1	
*Sp-EGFR1-A1*	*S. paramamosain*	Ov	Sp-EGFR^1^	MT663764.1
	*S. paramamosain*	Various	Sp-EGFR1^2^	WAR33937.1
*Sp-EGFR1-A2*	*S. paramamosain*	Various	Sp-EGFR2a^2^	WAR33938.1
*Sp-EGFR1-A3*	*S. paramamosain*	Various	Sp-EGFR2b^2^	WAR33939.1
*Sp-EGFR* ^3^	*S. paramamosain*	W	ScyparEVm000437t1*	
*Sp-EGFR* ^3^	*S. paramamosain*	W	ScyparEVm000437t2*	
*Sp-EGFR* ^3^	*S. paramamosain*	W	ScyparEVm000437t3*	

Contigs encoding EGFRs in the CrusTome 1.0 database and previously identified EGFRs in other decapods. Gene names are the proposed classification, based on clades and subclades from taxonomically comprehensive phylogenetic analyses. Species: *Gecarcinus lateralis, Carcinus maenas, Litopenaeus vannamei, Eriocheir sinensis, Cancer borealis*, and *Scylla paramamosain*. Tissue sources: CNS, central nervous system; MD, multiple developmental stages of whole larvae; N, neural tissues; Ov, ovary; PW, pooled whole organism; W, whole organism; and YO, Y-organ. Sequences available in **Supplementary Material 1**. Asterisk (*) indicates partial sequence; open reading frame incomplete.

^1^from (16). *Sp-EGFR* and *Sp-EGFR1* encode the same protein.

^2^from (17).

^3^Not assigned to isoforms.

**Figure 6:**
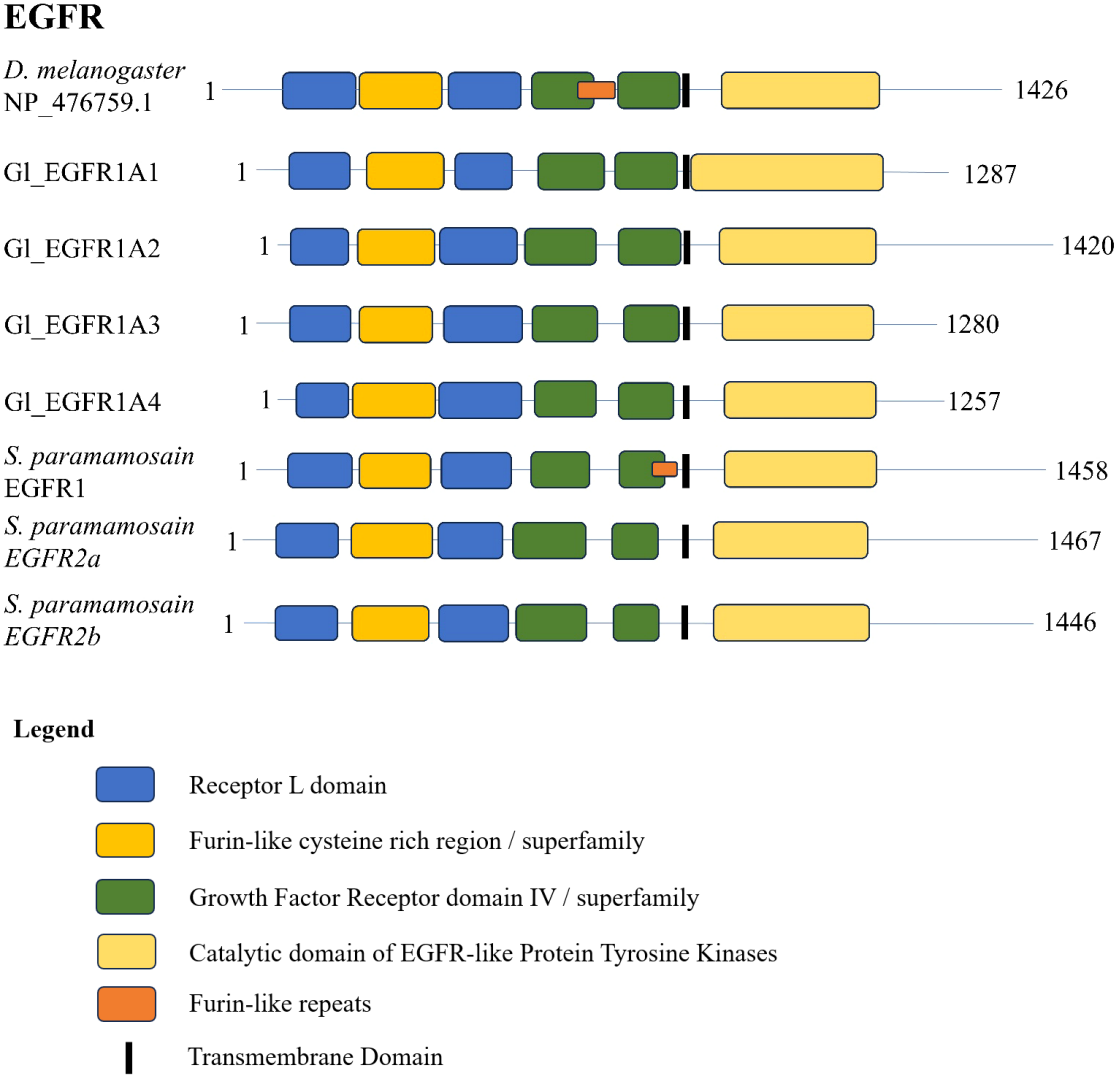
Domain organization of *Drosophila* and decapod EGF receptors. Listed sequences include a model organism (*D. melanogaster*), *G. lateralis* sequences, and identified genes in other species using the classification as listed in the original referenced studies (**Table 4**).

**Figure 7:**
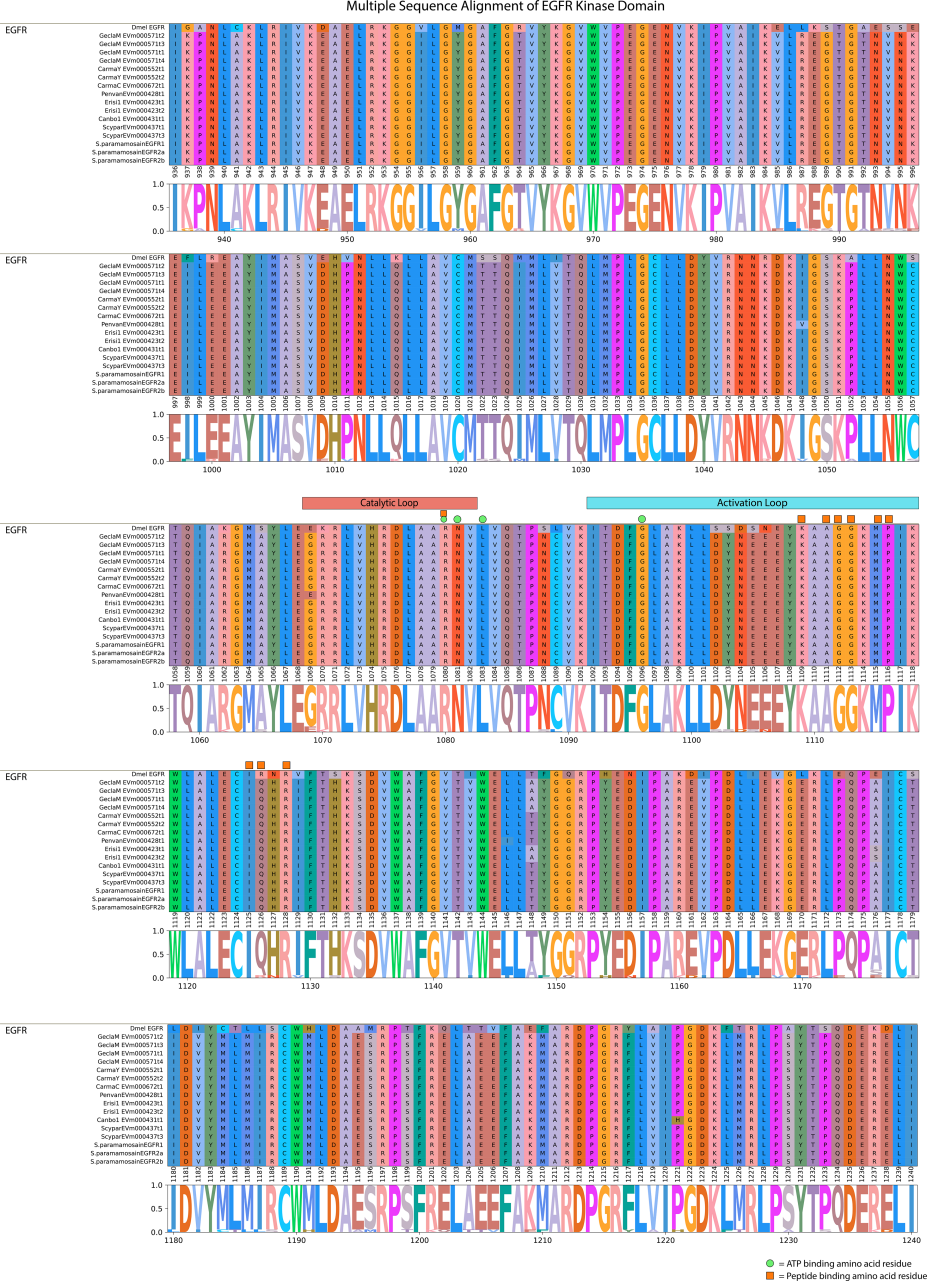
Multiple sequence alignment and logo plot of the catalytic domain of *Drosophila* and decapod EGF receptors. Includes representative species from **Table 4** (*Gecarcinus lateralis*, *Carcinus maenas, Cancer borealis, Litopenaeus vannamei, Scylla paramamosain*, and *Eriocheir sinensis*) and *Drosophila melanogaster* EGFR (Accession: NP476759.1) as a reference for comparison. The alignment illustrates the composition and length of conserved regions within subclades that reflect putative differences in ligands and/or binding affinities between receptor types. Catalytic loop and activation loop regions are demarcated by red and blue rectangles, respectively. ATP-binding and peptide-binding amino acid residues are annotated with green circles and orange squares, respectively, above the reference position. Partial sequences were excluded for ease of visualization and interpretation. MSA color scheme corresponds to similarities in physicochemical properties of amino acid residues. Logo plot illustrates conserved amino acid residues as a proportion of all the sequences included.

A conserved domain search for the *G. lateralis* and *S. paramamosain* EGFR sequences (17) and a *D. melanogaster* reference sequence showed a highly conserved domain organization. The N-terminal region contained two leucine repeat (Receptor L) domains, a furin-like cysteine rich region, and a growth factor receptor domain IV (**Figure 6**). The C-terminal region had an EGFR-like protein tyrosine kinase catalytic domain. The *D. melanogaster* sequence and Sp-EGFR1 sequence had an additional furin-like repeat (**Figure 6**). Multiple sequence alignment of the catalytic domain of the decapod EFGR with a *Drosophila* reference revealed high conservation of ATP-binding sites in the catalytic and activation loop regions (**Figure 7**). Ten peptide-binding residues were identified based on homology to *Drosophila*, nine of which were conserved across Decapoda and Hexapoda (**Figure 7**, reference alignment positions #1080, #1109, #1111, #1112, #1113, #1115, #1116, #1125, #1128). Only one peptide binding site differed between *Drosophila* and the decapods investigated (**Figure 7**, reference alignment position #1126), with both presenting hydrophilic residues (arginine and glutamine, respectively) in the aforementioned position.

### Crustacean fibroblast growth factor receptors

**Figure 8:**
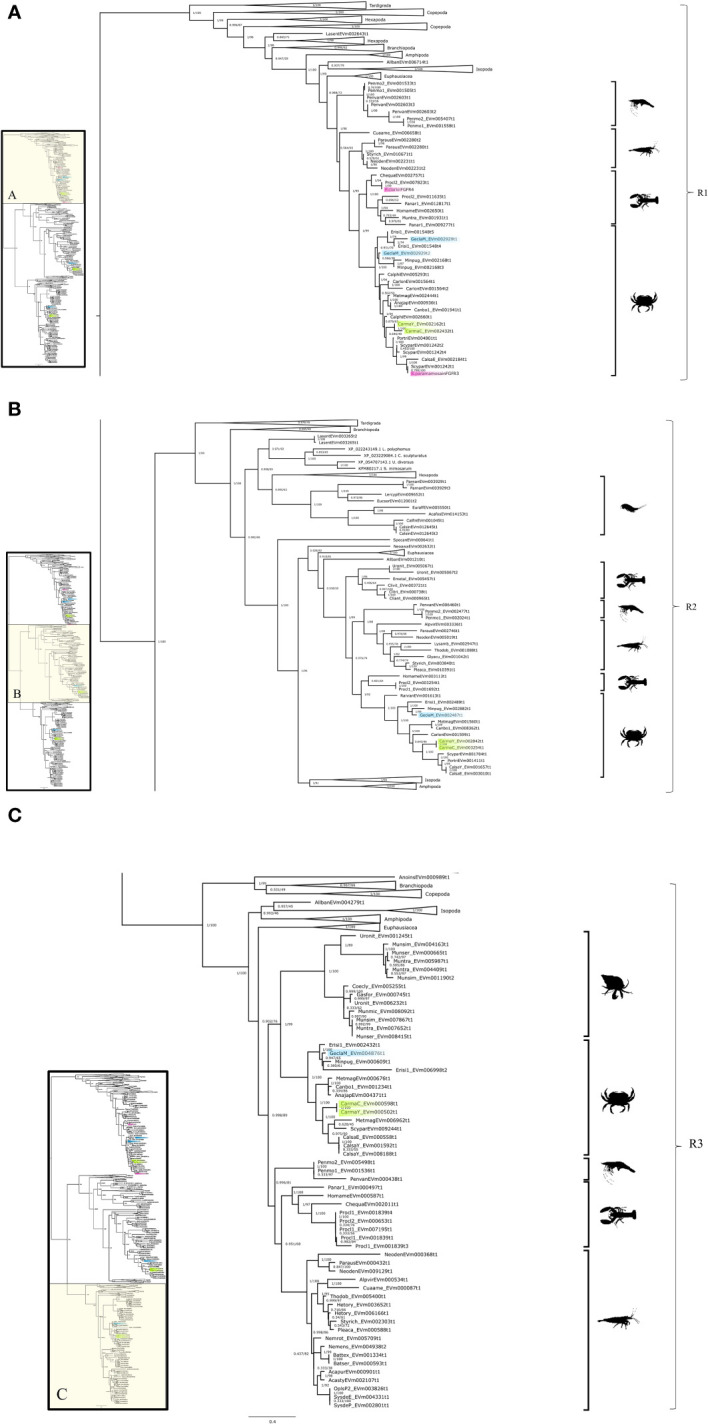
Fibroblast growth factor receptor phylogeny. A phylogenetic tree of FGFR consisting of three clades with a *G. lateralis* and *C. maenas* gene in each. Inset depicts entire tree, divided into sections **(A)** (FGFR1), **(B)** (FGFR2), and **(C)** (FGFR3), for orientation. Support values correspond to the approximate Bayes test and the Ultra-Fast Bootstrap approximation with the VT+F+R7 substitution model of evolution. Images from PhyloPic, as credited in **Figure 2**; *Copepoda* by Joel Vikberg Wernstrom.

Phylogenetic analysis of the CrusTome database identified three FGFR subclades, designated FGFR1, FGFR2, and FGFR3 (**Figure 8**). The three FGFR subclades included various pancrustacean taxonomic groups, with decapod species represented in all three subclades (**Figures 8A–C**).

Multiple FGFR contigs were identified in decapod species (**Figure 8**, **Table 5**). Analysis of *G. lateralis FGFR1* sequences and *C. maenas FGFR1*, *FGFR2*, and *FGFR3* sequences showed that the isoforms were products from a single gene for each subclade. In *G. lateralis*, two isoforms, designated *Gl-FGFR1-A1* and *-A2*, were apparently alternatively-spliced products of the same gene, based on highly conserved regions in the DNA alignment (**Table 5**, **Supplementary Material 2**). There were also two *C. maenas* isoforms of a single gene (*Cm-FGFR1-A1* and *Cm-FGFR1-A2*; **Table 5**, **Supplementary Material 2**). The FGFR2 and FGFR3 subclades had one *G. lateralis* contig sequence in each subclade (*Gl-FGFR2* and *Gl-FGFR3*) and two *C. maenas* isoform sequences in the FGFR2 subclade (*Cm-FGFR2-A1* and *Cm-FGFR2-A2*) (**Table 5**, **Supplementary Material 2**). Sequence alignment of *C. maenas* FGFR3 showed that the sequences were nearly identical, suggesting either allelic variation or slight discrepancies caused by the difference in tissue types (YO vs. CNS; **Table 5**).

**Table 5:** Classification of decapod FGF receptors.

Name	Species	Tissue	Transcript ID	Accession #
*Gl-FGFR1-A1*	*G. lateralis*	YO	GeclaM_EVm002929t1	OR772886
*Gl-FGFR1-A2*	*G. lateralis*	YO	GeclaM_EVm002929t2*	OR772887
*Cm-FGFR1-A1*	*C. maenas*	YO	CarmaY_EVm002162t1	OR772889
*Cm-FGFR1-A2*	*C. maenas*	CNS	CarmaC_EVm002432t1	OR772888
*Pc-FGFR1*	*P. clarkii*	He/Hp	*P. clarkii* FGFR4^1^	ON012066
*Sp-FGFR1*	*S. paramamosain*	He	*S. paramamosain* FGFR3^2^	ON045327
*Lv-FGFR1*	*L. vannamei*	P	PenvanEVm002603t1* PenvanEVm002603t2* PenvanEVm002603t3*	
*Es-FGFR1*	*E. sinensis*	MD	Erisi1_EVm001548t4 Erisi1_EVm001548t5	
*Cb-FGFR1*	*C. borealis*	N	Canbo1_EVm001941t1	
*Sp-FGFR1*	*S. paramamosain*	W	ScyparEVm001242t1 ScyparEVm001242t2 ScyparEVm001242t4	
*Gl-FGFR2*	*G. lateralis*	YO	GeclaM_EVm002487t1*	OR772890
*Cm-FGFR2-A1*	*C. maenas*	YO	CarmaY_EVm002842t1	OR772891
*Cm-FGFR2-A2*	*C. maenas*	CNS	CarmaC_EVm003254t1	OR772893
*Lv-FGFR2*	*L. vannamei*	P	PenvanEVm006460t1*	
*Es-FGFR2*	*E. sinensis*	MD	Erisi1_EVm002489t1	
*Cb-FGFR2*	*C. borealis*	N	Canbo1_EVm008362t1*	
*Sp-FGFR2*	*S. paramamosain*	W	ScyparEVm001704t1	
*Gl-FGFR3*	*G. lateralis*	YO	GeclaM_EVm004876t1*	OR772892
*Cm-FGFR3*	*C. maenas*	CNS	CarmaC_EVm000598t1	OR772895
	*C. maenas*	YO	CarmaY_EVm000502t1	OR772894
*Es-FGFR3*	*E. sinensis*	MD	Erisi1_EVm002432t1 Erisi1_EVm006998t2*	
*Cb-FGFR3*	*C. borealis*	N	Canbo1_EVm001234t1	
*Sp-FGFR3*	*S. paramamosain*	W	ScyparEVm009244t1*	
*Lv-FGFR3*	*L. vannamei*	P	PenvanEVm000438t1*	

Classification of contigs encoding decapod FGFRs in the CrusTome 1.0 database and previously identified FGFRs in other decapods. Classification was based on clades and subclades from taxonomically comprehensive phylogenetic analyses. Species: *Gecarcinus lateralis, Carcinus maenas, Procambarus clarkii, Litopenaeus vannamei, Eriocheir sinensis, Cancer borealis*, and *Scylla paramamosain*. Tissue sources: CNS, central nervous system; He, hemocytes; Hp, hepatopancreas; MD, multiple developmental stages of whole larvae; N, neural tissues; P, pooled whole organism; W, whole organism; and YO, Y-organ. Sequences available in **Supplementary Material 1**. Asterisk (*) indicates partial sequence; open reading frame incomplete.

^1^from (19).

^2^from (18).

**Figure 9:**
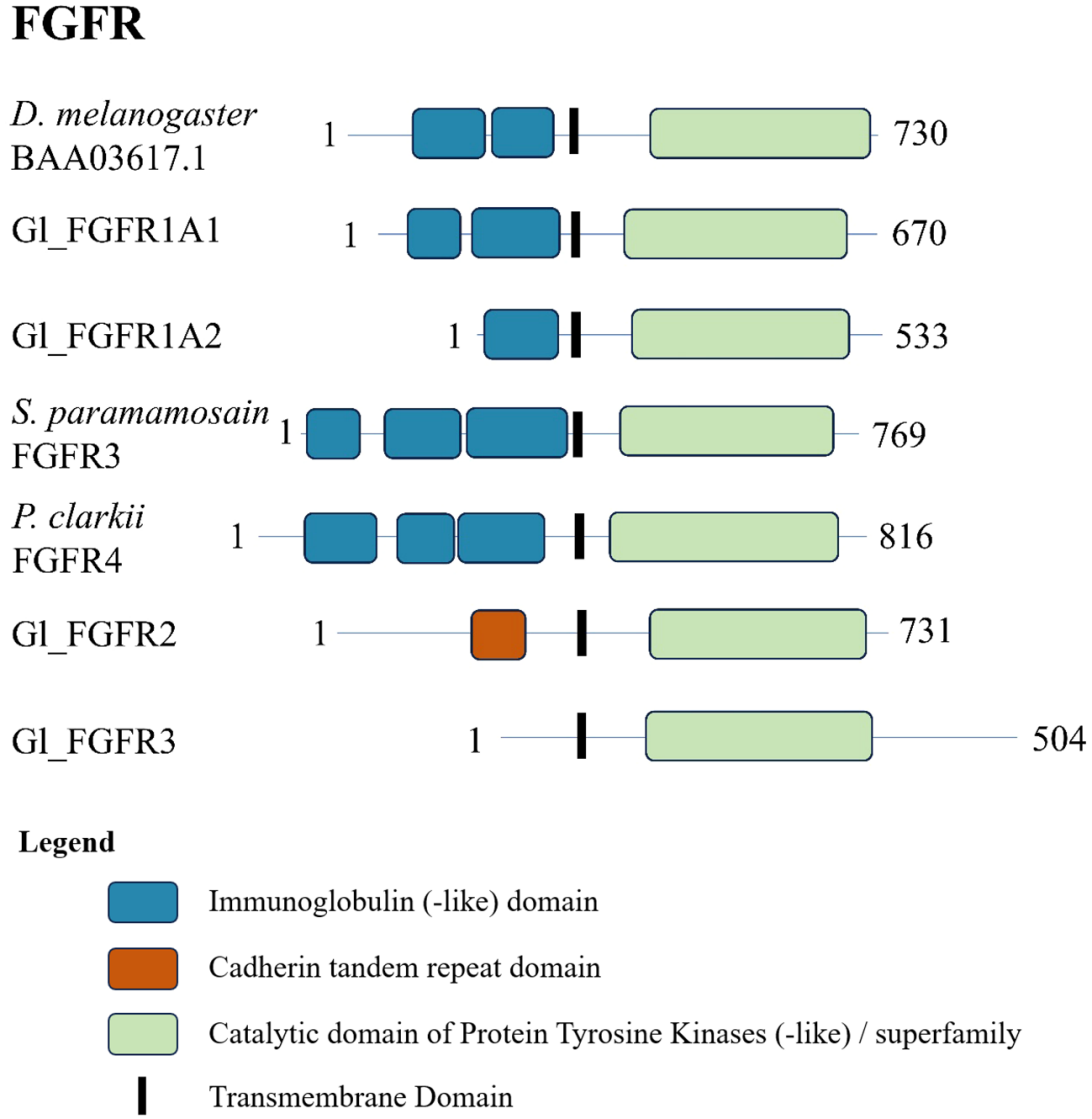
Domain organization of *Drosophila* and decapod FGF receptors. Listed sequences include a model organism (*D. melanogaster*), all *G. lateralis* sequences found, and identified genes in other species using the classification as listed in the original referenced studies (**Table 5**).

The decapod FGFR sequences showed a similar domain organization. Analysis of the *G. lateralis* Gl-FGFR1-A1 sequence, the Sp-FGFR3 and Pc-FGFR4 sequences from previous studies (18, 19), and a *D. melanogaster* reference FGFR sequence showed two to three immunoglobulin-like domains in the N-terminal region and a protein tyrosine kinase catalytic domain in the C-terminal region (**Figure 9**). Gl-FGFR1-A2 was a partial sequence missing a portion of the N-terminal sequence; only one immunoglobulin-like domain was identified (**Figure 9**). Gl-FGFR2 and Gl-FGFR3 were partial sequences that lacked immunoglobulin domains (**Table 5**, **Figure 9**). Interestingly, the N-terminus of Gl-FGFR2 had a cadherin tandem repeat domain (**Figure 9**).

**Figure 10:**
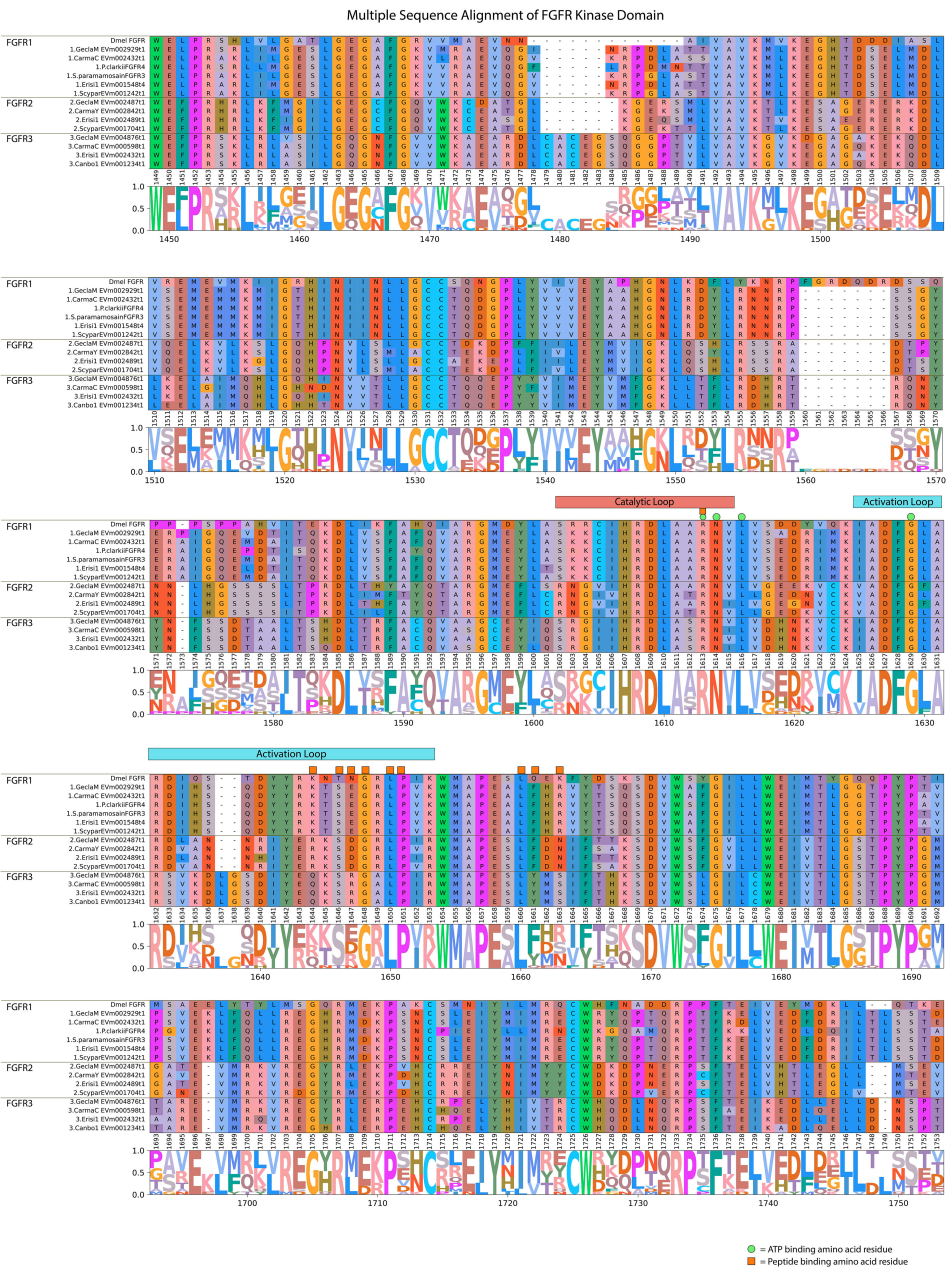
Multiple sequence alignment and logo plot of the catalytic domain of *Drosophila* and decapod FGF receptors. Includes representative species from **Table 5** (*Gecarcinus lateralis*, *Carcinus maenas*, *Procambarus clarkii, Eriocheir sinensis, Cancer borealis*, and *Scylla paramamosain*) and *Drosophila melanogaster* FGFR (Accession: BAA03617.1) as a reference for comparison. The alignment illustrates the composition and length of conserved regions within subclades that reflect putative differences in ligands and/or binding affinities between receptor types. Catalytic loop and activation loop regions are demarcated by red and blue rectangles, respectively. ATP-binding and peptide-binding amino acid residues are annotated with green circles and orange squares, respectively, above the reference position. Partial sequences were excluded for ease of visualization and interpretation. MSA color scheme corresponds to similarities in physicochemical properties of amino acid residues. Logo plot illustrates conserved amino acid residues as a proportion of all the sequences included.

MAGA search and a multiple sequence alignment of the FGFR contigs in **Table 5** identified a motif in the catalytic domain that distinguished the three decapod FGFR subclades (**Table 6**) (67). The 113-amino acid sequence in FGFR1 and 112-amino acid sequences in FGFR2 and FGFR3 were bounded by a conserved “VAVK” at the N-terminal end and a conserved “HRDLA” at the C-terminal end (**Figure 10**; reference alignment positions #1492-1495 and #1606-1611, respectively). Moreover, multiple sequence alignments of the catalytic domain of decapod FGFRs with a *Drosophila* reference revealed amino acids in the ATP-binding and peptide-binding sites that distinguished the decapod FGFRs. The four residues for ATP binding within the catalytic loop and activation loop regions were completely conserved in decapod and *Drosophila* FGFRs (**Figure 10**; reference alignment positions #1613, #1614, #1616, and #1629). Six of the ten peptide-binding residues were completely conserved in all the decapod FGFRs (**Figure 10**; reference alignment positions #1613, #1646, #1648, #1650, #1651, and #1660). Interestingly, the other four residues were conserved within each of the three subtypes (**Figure 10**, reference alignment positions #1644, #1647, #1661, and #1663). Specifically, at position #1644, the residues in FGFR1, FGFR2, and FGFR3 were lysine (K), arginine (R), or glutamine (Q), respectively. At position #1647, the residues in FGFR1, FGFR2, and FGFR3 were glutamate (E), aspartate (D), or R, respectively. At position #1661, the residues in FGFR1, FGFR2, and FGFR3 were phenylalanine (F), F, or tyrosine (Y), respectively. At position #1663, the residues in FGFR1, FGFR2, and FGFR3 were R, asparagine (N), or methionine (M), respectively.

**Table 6:** Motif sequences distinguishing the three decapod FGFRs.

Receptor	Consensus sequences
FGFR1	**VAVK**ML**K**EGHTDx**E**LM**DL**VS**E**MEMMKMI**G**THI**N**IINL**L**G**CC** **TQDGP**LYVVV**EY**AAH**G**N**L**RDY**LR**Nx**R**xxSG**Y**ERxIGQExxxxxx x**DL**VSFxx**Q**VAR**G**MEYLxSxKCI **HRDLA** A **RN** V **L**
FGFR2	**VAVK**Tx**K**ESAxxR**E**Rx**DL**VQ**E**LKVLKxL**G**xHx**N**VxSx**L**x **CC**xxKx**P**xFxxL**EY**Mxx**G**K**L**QSx**LR**xS**R**ADTx**Y**xN-LHGSSSSxTPx**DL**xxxxY**Q**xxR**G**MEFLxRNxxx **HRDLA** x **RN** x **L**
FGFR3	**VAVK**GV**K**xGAGxK**E**KQ**DL**Lx**E**LxIMQHx**G**xxx**N**VVTL**L** G**CC**TQQE**P**xxVIM**EY**VMF**G**K**L**LxF**LR**DH**R**TRxN**Y**YN-FSSDTxALTSx**DL**TRFAC**Q**VAx**G**CEYxQSRGII **HRDLA** x **RN** x **L**

FGFR1, FGFR2, and FGFR3 were distinguished by a 118-amino acid motif sequence in FGFR1 and by 117-amino acid sequences in FGFR2 and FGFR3, located in the catalytic domain in the C-terminal region. The 16-amino acid catalytic loop is underlined. Residues that are identical between all the sequences from **Table 5** are indicated by bold font. Consensus sequences were obtained using the MAGA tool (67) and multiple sequence alignment (**Figure 10**).

### Crustacean vascular endothelial and platelet-derived growth factor receptors (PVRs)

**Figure 11:**
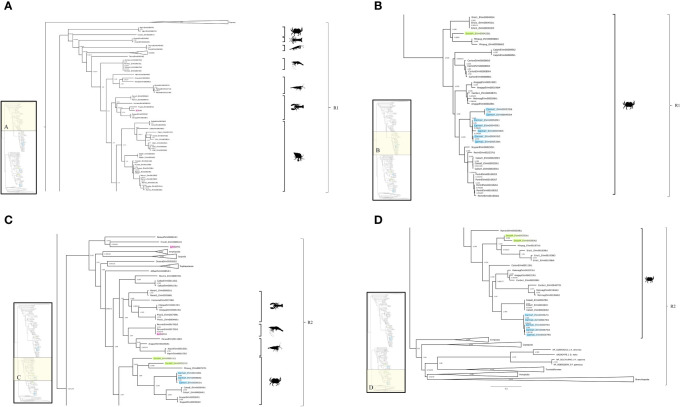
PDGFR/VEGFR-related receptor (PVR) phylogeny. A phylogenetic tree of PVR of four potential *G. lateralis* genes and three *C. maenas* genes. Inset depicts entire tree, divided into sections **(A)** (PVR1), **(B)** (PVR1), and **(C)** (PVR2), and **(D)** (PVR2). Support values shown correspond to the approximate Bayes test and the Ultra-Fast Bootstrap approximation with the WAG+F+I+I+R7 substitution model of evolution. Images from PhyloPic, as credited in **Figure 2**; *Penaeus monodon* (tiger prawn) by T. Michael Keesey; *Squilla mantis* (mantis shrimp) by T. Michael Keesey; and *Euphausiidae* (krill) by Steven Haddock.

Initially, VEGFR and PDGFR phylogenetic trees were created separately (**Supplementary Material 2**). BLAST searches identified the same sequences in both trees, indicating that crustacean VEGFRs and PDGFRs constituted a single RTK class. Consequently, a phylogenetic analysis was conducted on a single group, designated PDGF/VEGF-related receptors (PVRs). Phylogenetic analysis identified three well-supported subclades that further segregated along taxonomic lineages (**Figure 11**). BLAST searches of the outgroup subclade identified sequences as low-density lipoprotein receptors (**Figure 11A**). The remaining two subclades were designated PVR1 and PVR2 (**Figure 11**). PVR1 included sequences from Euphausiacea, Stomatopoda, and Decapoda (Anomura, Astacidea, Brachyura, Caridea, and Dendrobranchiata) (**Figures 11A, B**). PVR2 included sequences from diverse pancrustacean taxa (e.g., Amphipoda, Branchiopoda, Cirripedia, Copepoda, Decapoda, Euphausiacea, Hexapoda, and Isopoda) (**Figures 11C, D**).

Several *PVR1* contig sequences were identified in decapods (**Table 7**). One *C. maenas* gene was identified with four isoforms, designated *Cm-PVR1-A1, -A2, -A3*, and *-A4* (**Figure 11B**, **Table 7**, **Supplementary Material 2**). Two contigs are listed for each of the *Cm-PVR1-A1*, *-A2*, and *-A4* isoforms, as they had small variations that may be due to different tissue sources (**Table 7**, **Supplementary Material 2**). Only one *PVR1* contig sequence was identified in *G. lateralis*, *C. borealis*, *S. paramamosain*, and *Pacifasticus leniusculus* (**Table 7**).

**Table 7:** Classification of decapod PDGF/VEGF-related receptors (PVRs).

Name	Species	Tissue	Transcript ID	Accession #
*Gl-PVR1*	*G. lateralis*	YO	GeclaM_EVm000425t1	OR772896
*Cm-PVR1-A1*	*C. maenas*	CNS YO	CarmaC_EVm000535t6 CarmaY_EVm000455t4*	OR772898 OR772897
*Cm-PVR1-A2*	*C. maenas*	CNS YO	CarmaC_EVm000535t1 CarmaY_EVm000455t1	OR772899 OR772911
*Cm-PVR1-A3*	*C. maenas*	YO	CarmaY_EVm000455t5	OR772912
*Cm-PVR1-A4*	*C. maenas*	CNS YO	CarmaC_EVm000535t4 CarmaY_EVm000455t3	OR772914 OR772913
*Cb-PVR1*	*C. borealis*	N	Canbo1_EVm002684t1*	
*Es-PVR1*	*E. sinensis*	MD	Erisi1_EVm000492t1* Erisi1_EVm000492t3* Erisi1_EVm000492t4	
*Cb-PVR1*	*C. borealis*	N	Canbo1_EVm000387t1*	
*Sp-PVR1*	*S. paramamosain*	W	ScyparEVm000253t1	
*Pl-PVR1*	*P. leniusculus*	HeTC	Pl_PVR1^1^	KY444650
*Gl-PVR2-A1*	*G. lateralis*	YO	GeclaM_EVm000511t1	OR772915
*Gl-PVR2-A2*	*G. lateralis*	YO	GeclaM_EVm000521t1	OR772917
*Cm-PVR2-A1a*	*C. maenas*	CNS YO	CarmaC_EVm001015t1* CarmaY_EVm000863t2*	OR772916 OR772918
*Cm-PVR2-A1b*	*C. maenas*	YO	CarmaY_EVm000863t1	OR772920
*Lv-PVR2-A*	*L. vannamei*	Various Various PW PW	LvVEGFR1^2^ LvVEGFR2^2^ PenvanEVm001782t4* PenvanEVm001782t5	KM280384 MF417824
*Sp-PVR2-A*	*S. paramamosain*	W	ScyparEVm000304t1 ScyparEVm000304t3	
*Gl-PVR2-B1a*	*G. lateralis*	YO	GeclaM_EVm000503t1	OR772919
*Gl-PVR2-B1b*	*G. lateralis*	YO	GeclaM_EVm000503t2*	OR772921
*Cm-PVR2-B1a*	*C. maenas*	YO CNS	CarmaY_EVm000567t1 CarmaC_EVm000679t1	OR772922 OR772923
*Cm-PVR2-B1b*	*C. maenas*	CNS	CarmaC_EVm000679t4*	OR772925
*Cm-PVR2-B1c*	*C. maenas*	CNS	CarmaC_EVm000679t3*	OR772924
*Cm-PVR2-B1d*	*C. maenas*	CNS	CarmaC_EVm000679t5*	OR772926
*Es-PVR2-B*	*E. sinensis*	MD	Erisi1_EVm001938t1 Erisi1_EVm001938t3* Erisi1_EVm001938t6*	
*Cb-PVR2-B*	*C. borealis*	N	Canbo1_EVm000508t1 Canbo1_EVm004877t1	

Contigs encoding PVRs in the CrusTome 1.0 database and previously identified PVRs in other decapods. Gene names are the proposed classification, based on clades and subclades from taxonomically comprehensive phylogenetic analyses. The *C. maenas* sequences with the same classification are the same version of a gene/isoform from different tissues with the small differences in sequences. Species: *Gecarcinus lateralis, Carcinus maenas, Pacifastacus leniusculus, Cancer borealis, Eriocheir sinensis, Litopenaeus vannamei*, and *Scylla paramamosain*. Tissue sources: CNS, central nervous system; HeTC, Hematopoietic Tissue Cells; N, neural tissues; MD, multiple developmental stages of whole larvae; PW, pooled whole organism; W, whole organism; and YO, Y-organ. Sequences available in **Supplementary Material 1**. Asterisk (*) indicates partial sequence; open reading frame incomplete.

^1^from (29).

^2^from (70, 71).

The decapod *PVR2* sequences were separated into two well-supported groups, designated *PVR2-A* and *PVR2-B* (**Figures 11C, D**, **Table 7**). Two *G. lateralis* contigs, designated *Gl-PVR2-A1* and *Gl-PVR2-A2*, differed in single nucleotide polymorphisms, suggesting that they were products of two genes (**Table 7**, **Supplementary Material 2**). By contrast, two *C. maenas* isoforms of one gene were identified (*Cm-PVR2-A1a* and *Cm-PVR2-A1b*; **Table 7**). Two *C. maenas* contigs, one from CNS and the other from YO, were assigned to *Cm-PVR2-A1a*, due to their high similarity in sequence identity (**Table 7**, **Supplementary Material 2**). Four sequences were assigned to *Lv-PVR2-A* and two sequences were assigned to *Sp-PVR2-A* without further analysis (**Table 7**). In the *PVR2-B* group, *G. lateralis* had two isoforms from one gene, designated *Gl-PVR2-B1a* and *-B1b*, and *C. maenas* had four isoforms from one gene, designated *Cm-PVR2-B1a, -B1b, -B1c*, and *-B1d* (**Table 7**, **Supplementary Material 2**). Two contigs, obtained from YO and CNS transcriptomes, were assigned to *Cm-PVR2-B1a*, as the contigs had high sequence identity (**Table 7**, **Supplementary Material 2**). Three contig sequences were assigned to *Es-PVR2-B* and two sequences were assigned to *Cb-PVR2-B* without further analysis (**Table 7**).

**Figure 12:**
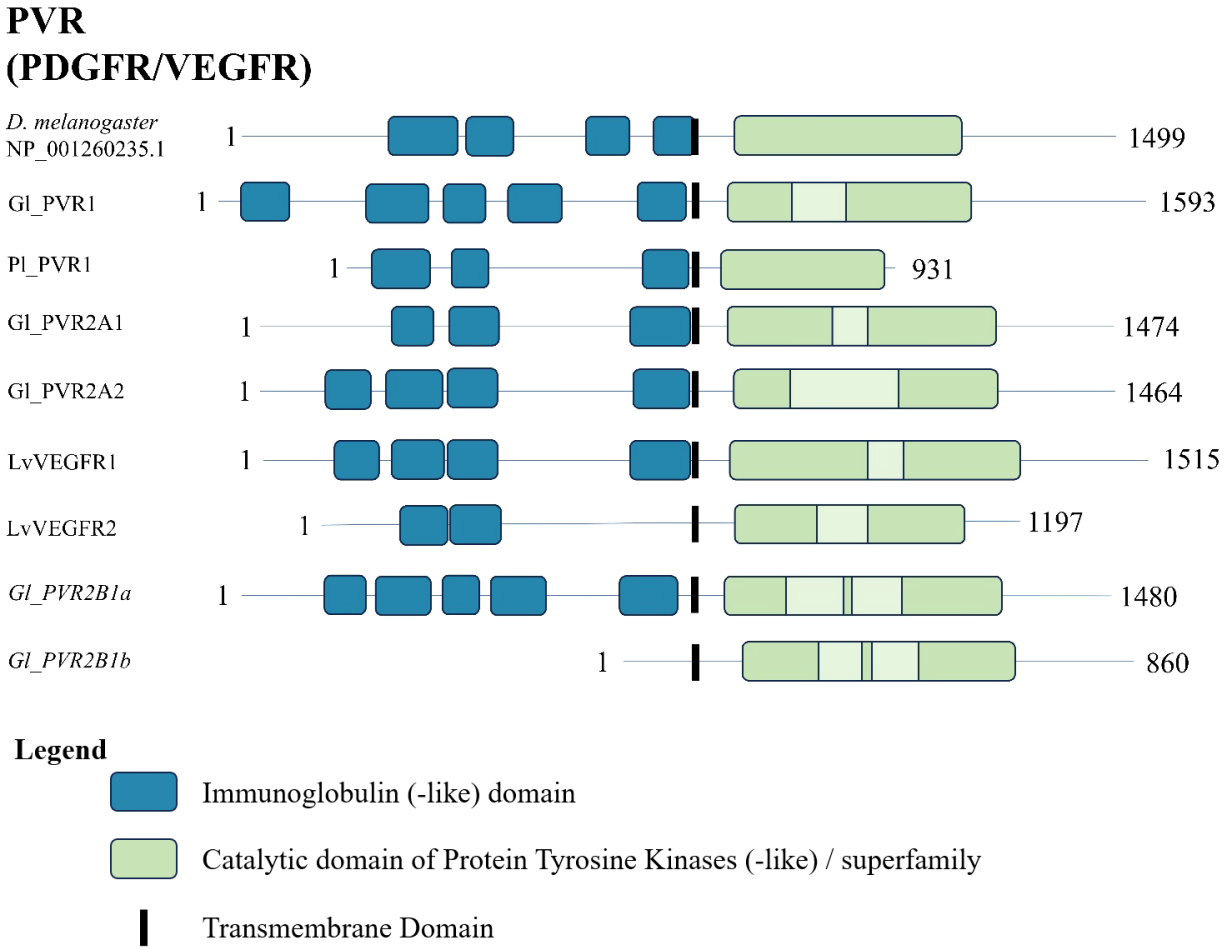
Domain organization of PDGF/VEGF (PV) receptors. Listed sequences include a model organism (*D. melanogaster*), all *G. lateralis* sequences found, and identified genes in other species using the classification as listed in the original referenced studies (**Table 7**). The shaded light green sections are the kinase-insert domains in mammalian PDGFRs and VEGFRs (6).

A conserved domain search of the *G. lateralis*, *L. vannamei*, and *P. leniusculus* PVR sequences and a *D. melanogaster* reference sequence revealed a similar domain organization. The N-terminal region had between two and five immunoglobulin-like domains (**Figure 12**). The C-terminal region had a protein tyrosine kinase catalytic domain (**Figure 12**).

**Figure 13:**
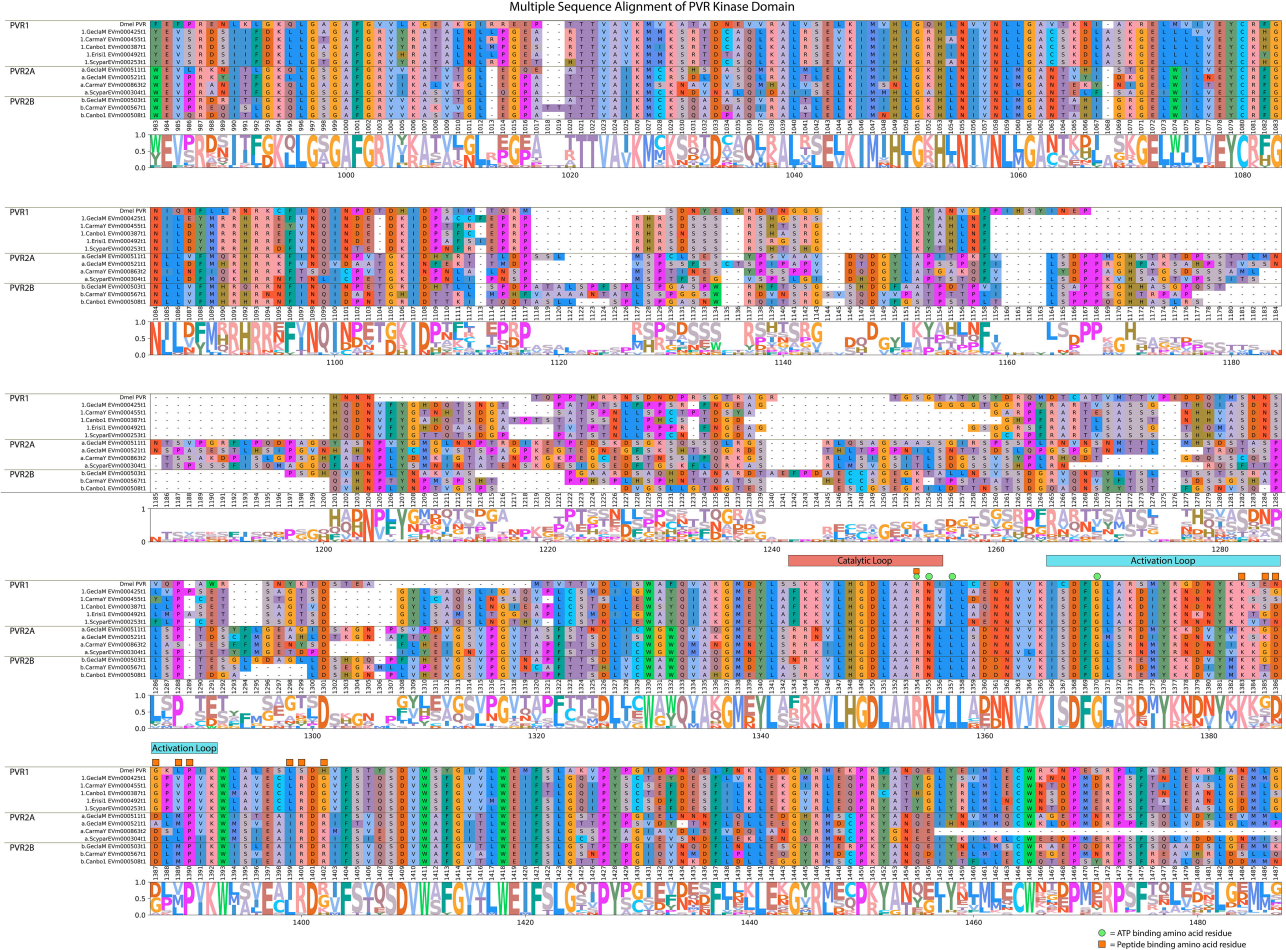
Multiple sequence alignment and logo plot of the catalytic domain of *Drosophila* and decapod PDGF/VEGF (PV) receptors. Includes representative species from **Table 7** (*Gecarcinus lateralis*, *Carcinus maenas*, *Cancer borealis, Eriocheir sinensis*, and *Scylla paramamosain*) and *Drosophila melanogaster* PVR (Accession: NP001260235.1) as a reference for comparison. The alignment illustrates the composition and length of conserved regions within subclades that reflect putative differences in ligands and/or binding affinities between receptor types. Catalytic loop and activation loop regions are demarcated by red and blue rectangles, respectively. ATP-binding and peptide-binding amino acid residues are annotated with green circles and orange squares, respectively, above the reference position. Partial sequences were excluded for ease of visualization and interpretation. MSA color scheme corresponds to similarities in physicochemical properties of amino acid residues. Logo plot illustrates conserved amino acid residues as a proportion of all the sequences included.

MAGA search and a multiple sequence alignment of the contigs in **Table 7** identified a 46-amino acid motif in the catalytic domain that distinguished the PVR1 and PVR2 subclades (**Table 8**) (67). The motif was bounded a conserved “HGDLA” at the N-terminal end and a conserved “PxKW” at the C-terminal end (**Table 8**, **Figure 13**, reference alignment positions #1348-1352 and #1390-1393, respectively). It should be noted that the glycine (G) in the HGDLA sequence was replaced by an arginine (R) in Lv-VEGFR2 (**Supplementary Material 2**) (70). The PVR motif was located N-terminal to the FGFR motif, with the HGDLA/HRDLA sequence marking the N-terminal and C-terminal boundaries of the PVR and FGFR motifs, respectively (**Tables 6**, **8**). Multiple sequence alignment of the catalytic domain of decapod PVRs with a *Drosophila melanogaster* reference revealed structural diversity between and within PVR subtypes (**Figure 13**). The four amino acids identified in the ATP-binding site in the catalytic and activation loop region were completely conserved (**Figure 13**; reference alignment positions #1354, #1355, #1357, and #1370). By contrast, only four of the ten peptide-biding residues were completely conserved in decapod PVRs (**Figure 13**, reference alignment positions #1354, #1383, #1390, and #1400). Analogous to the FGFRs, four of the other six peptide-binding residues were conserved between the three PVR subtypes (**Figure 13**, reference alignment positions #1387, #1389, #1399, and #1402). Specifically, at position #1387, PVR1, PVR2A, and PVR2B had glycine (G), aspartate (D) or alanine (A), or D (**Figure 13**). At position #1389, PVR1, PVR2A, and PVR2B had valine (V), methionine (M) or leucine (L), or M, respectively. At position #1399, PVR1, PVR2A, and PVR2B had L, isoleucine (I), or I, respectively. At position #1402, PVR1, PVR2A, and PVR2B had G, arginine (R) or lysine (K), or R, respectively. The residues at positions #1385 and #1386 were more variable (**Figure 13**).

**Table 8:** Motif sequences distinguishing the decapod PVRs.

Receptor	Consensus sequences
PVR1	Y**Q**I**A**K**GM**E**YL** AFKKVL**HGDLA**A**RN**V **LL**xx N**N**VV**KISDFG** LAKDI **Y** xNxN **Y** K **K** xxxGPV **P** V **KW**
PVR2A	W**Q**x**A**x**GM**x**YL** SRRxxL **HGDLA** A **RN** L **LL**x DN**N**Vx**KISDFG** xSRxx **Y** xxxx **Y** x **K ** xxDxxx **P ** x **KW**
PVR2B	W**Q**V**A**x**GM**x**YL** xxRKVL **HGDLA** A **RN** L **LL**xDD**N**xx **KISDFG ** LSRxM **Y** KKDx **Y** M **K** KxDDLM **P** I **KW**

PVR1, PVR2A, and PVR2B were distinguished by 62-amino acid motif sequences spanning the catalytic and activation loops in the catalytic domain in the C-terminal region. Catalytic loop indicated by double underline and activation loop indicated by dashed underline. Residues that are identical between all the sequences from **Table 7** are indicated by bold font. Consensus sequences were obtained using the MAGA tool (67) and multiple sequence alignment (**Figure 13**).

## Discussion

Phylogenetic analysis of the CrusTome database yielded the most extensive catalog of Pancrustacea RTK contig sequences to date. The large number of species from major Crustacea taxa provided a higher confidence in distinguishing RTK types and identifying genes and isoforms. A total of 988 contigs encoding RTKs in the CrusTome database were identified in 118 crustacean species, 36 in three hexapod species, and nine in two tardigrade species (**Table 1**, **Supplementary Material 1**). The sequences segregated into well-supported clades and subclades, which formed the basis for their classification into RTK types and subtypes (**Figure 1**). InsR and EGFR were sister clades, as they shared furin-like repeat and leucine-rich repeat (Receptor L) domains in the N-terminal region (**Figures 1**, **3**, **6**) (6–8, 72). FGFR and PVR were clustered together, as they had immunoglobulin domains in the N-terminal region (**Figures 1**, **9**, **12**) (6, 9, 11, 72). This is consistent with the inferred evolutionary histories with the ancestral versions being and/or containing InsR and EGFR domains, and FGFR, PDGFR, and VEGFR constituting later evolved receptors (72, 73). Interestingly, while the other receptors distributed into a number of subclades, EGFR was highly conserved across crustacean taxa, suggesting that its role in physiological processes is conserved across the Metazoa. RTK subclades often contained diverse pancrustacean taxa, and their topologies mirrored pancrustacean evolutionary history (74). This suggests that ancient duplication events gave rise to the diversity of RTKs observed today (31). In addition, the aforementioned phylogenetic reconstructions and classification resulted in a high diversity of newly characterized arthropod RTK sequences.

Sixty decapod InsR sequences were organized into three subtypes, designated InsR1, InsR2, and InsR3 (**Figure 1**, **Table 1**). *InsR1* contigs were identified in *G. lateralis*, *C. maenas*, *E. sinensis*, and *C. borealis* transcriptomes (**Table 2**). *InsR2* contigs were identified in *C. maenas*, *C. borealis*, *S. paramamosain* (*Sp-IR*), *F. chinensis* (*Fc-IAGR*), and *S. verreauxi* (*Sv-TKIR*) (**Figure 2**, **Table 2**, **Supplementary Material 2**). *InsR3* contigs were identified in *G. lateralis* (3 isoforms), *C. maenas* (2 isoforms), *M. rosenbergii* (*Mr-IR*), and *C. borealis* (**Figure 2**, **Table 2**, **Supplementary Material 2**). *Gl-InsR1* contained a nucleotide sequence of 1264 bp; *Gl-InsR3-A1*, *-A2*, and *-A3* contained nucleotide sequences of 5647 bp, 2530 bp, and 2206 bp, respectively. The *de novo* assemblies produced only partial sequences, possibly due to low levels of expression in the sequenced tissues. A full-length *Gl-InsR3-A1* sequence was constructed from three overlapping partial contigs (**Table 2**). *Gl-InsR3-A1* was similar to cDNAs encoding *M. rosenbergii* insulin receptor (Mr-IR) and *E. sinensis* insulin-like receptor (Es-InR); the sequences were assigned to the R3 subclade (**Table 2**, **Supplementary Material 2**) (21, 23). An InsR binds insulin-like androgenic gland hormone (IAG), an insulin-like peptide (ILP) that determines male sexual characters by the androgenic gland (75–77). *InsR2* subclade members in *S. paramamosain*, *S. verreauxi*, *L. vannamei*, and *F. chinensis* (**Table 2**) appear to be IAG receptors, as *InsR2* is only expressed in male reproductive tissues (e.g., testis, sperm duct, terminal ampullae, and androgenic gland) and RNAi knockdown of InsR2 reduces testicular development (25, 27). Moreover, *in vitro* binding assays show interactions between IAG and InsR2 (25, 26). The ligands of the InsR1 and InsR3 subclades are unknown. dsRNA knockdown of *Mr-IR*/*Mr-InsR-R3* did not result in sex reversal, suggesting that a different InsR gene is involved (21). However, Tan et al. (2020) reported sex reversal in one or two *M. rosenbergii* individuals with dsRNA or siRNA knockdown of *Mr-IR* (22). *Es-InR*/*Es-InsR3* is implicated in limb regeneration, as *Es-InR* is up-regulated in limb regenerates and an InR inhibitor (GSK1838705A) suppresses limb regenerate growth (23).

Seventy-seven decapod EGFR sequences were organized into a single monophyletic clade (EGFR1; **Figure 1**, **Table 1**). The assignment of the EGFR1s to a single clade was supported by the high amino acid sequence identity in the catalytic domain (**Figure 7**). Multiple isoforms were common (**Figure 5**, **Table 4**). *G. lateralis* had one EGFR gene with four isoforms obtained from the YO transcriptome; the contigs ranged from 4280 bp to 5550 bp and classified as *Gl-EGFR1-A1*, *-A2*, *-A3*, and *-A4* (**Figure 5**, **Table 4**). Four *C. maenas* EGFR isoforms were also obtained - two from the YO transcriptome and two from the CNS transcriptome (**Figure 5**, **Table 4**). This compares to a single 6864-bp *M. rosenbergii* EGFR sequence obtained from the SRA database (**Table 4**) (20). Single EGFR sequences were also obtained from *L. vannamei*, *E. sinensis*, and *C. borealis* transcriptomes (**Table 4**). Three distinct EGFR transcripts varying between 5076 bp and 5457 bp have been identified in *S. paramamosain* (**Table 4**) (17). cDNAs encoding two genes, designated *Sp-EGFR1* and Sp-*EGFR2*, were obtained by PCR of genomic DNA, followed by RACE of RNA from hepatopancreas (17). *Sp-EGFR1* produces a single coding sequence, whereas *Sp-EGFR2* produces two alternatively-spliced isoforms, designated *Sp-EGFR2a* and *Sp-EGFR2b* (17). Previously, a full-length *Sp-EGFR* sequence was cloned from ovary (16). As the protein sequences of Sp-EGFR and Sp-EGFR1 are identical, it is likely that they are products of the same gene. Comprehensive phylogenetic analyses and multiple sequence alignments in the present study suggest that all three sequences are isoforms of one gene and not two separate gene products as previously hypothesized by Cheng et al. (17). Three partial contig sequences identified in the CrusTome database matched the three *S. paramamosain* cDNA sequences (**Table 4**, **Figure 5**, **Supplementary Material 2**). Thus, there are three *Sp-EGFR* coding sequences, which are designated *Sp-EGFR1-A1*, *-A2*, and *-A3* (**Table 4**).

Decapod EGFRs, which are widely expressed in tissues, mediate physiological processes involving growth and differentiation. *Mr-EGFR* is expressed in thoracic ganglion, heart, hepatopancreas, muscle, ovary in females, and testis and sperm duct in males (20). dsRNA knockdown of *Mr-EGFR* in male prawns inhibits molt-incremental growth; inhibits growth of a male-specific secondary sexual characteristic (appendix masculina); and disrupts eye ommatidia organization (20). In *S. paramamosain*, *EGFRs* are expressed in all tissues (16, 17). *Sp-EGFR*/*Sp-EGFR1-A1* is expressed in 14 tissues, with higher expression in heart, YO, ovary, gill, and stomach (16). *Sp-EGFR1*/*Sp-EGFR1-A1*, *Sp-EGFR2a*/*Sp-EGFR1-A2*, and *Sp-EGFR2b*/*Sp-EGFR1-A3* are expressed in 8 tissues (17). *Sp-EGFR1*/*Sp-EGFR1-A1* and *Sp-EGFR2a*/*Sp-EGFR1-A2* are expressed at higher levels than *Sp-EGFR2b*/*Sp-EGFR1-A3* in gill, hepatopancreas, ganglion, stomach, and muscle (17). Sp-EGFR signaling promotes ovarian development. *Sp-EGFR* mRNA levels increase in early and late vitellogenic stages (16). Human EGF stimulates vitellogenesis and *Sp-Vitellogenin receptor* expression in oocytes *in vitro*, which is inhibited by EGFR inhibitors AG1478 and PD153035 (16).

One hundred and twenty-nine decapod FGFR sequences were organized into three clades, designated FGFR1, FGFR2, and FGFR3 (**Figure 1**, **Table 1**). FGFR1 contigs were identified in *G. lateralis* (2 isoforms), *C. maenas* (2 isoforms), *L. vannamei*, *E. sinensis*, *C. borealis*, and *S. paramamosain* (**Table 5**). A cDNA encoding Sp-FGFR3 was cloned from *S. paramamosain* hemocytes (18). As the *Sp-FGFR1* contig sequences and the *Sp-FGFR3* sequence were similar (**Figure 8A**), *Sp-FGFR3* was assigned to the FGFR1 subtype (*Sp-FGFR1*; **Table 5**). Likewise, a cDNA encoding Pc-FGFR4, which was cloned from *P. clarkii* hemocytes and hepatopancreas (19), clustered with other decapod FGFR1 sequences (designated *Pc-FGFR1*; **Figure 8A**). Gl-FGFR1 proteins with less than three immunoglobulin domains were partial sequences (**Figure 9**). FGFR2 and FGFR3 contigs were identified in *G. lateralis*, *C. maenas* (2 isoforms), *L. vannamei*, *E. sinensis*, *C. borealis*, and *S. paramamosain*; all seven *FGFR2* and all seven *FGFR3* sequences were novel (**Table 5**). The N-terminal region of Gl-FGFR2 and Gl-FGFR3 lacked immunoglobulin domains (**Figure 9**). Interestingly, Gl-FGFR2 had a cadherin tandem repeat domain, which occurs in other RTKs (78). This illustrates the challenge of using sequence-similarity based methods for growth factor receptor identification. However, their identity as FGFRs was confirmed by the conserved protein tyrosine kinase domain shared by all the decapod sequences (**Figures 9**, **10**).

There are few reports on the functions of FGFRs in decapods, and those studies are restricted to members of the FGFR1 subclade. The functions of FGFR2 and FGFR3 are unknown. In *P. clarkii* and *S. paramamosain*, FGFR1 is involved in innate immunity. Viral and bacterial infection increases mRNA levels of *Sp-FGFR3* in the hepatopancreas and *Pc-FGFR4* in hemocytes and hepatopancreas (18, 19). Moreover, RNAi knockdown of *Pc-FGFR4* and *Sp-FGFR3* or FGFR inhibitor (Pemigatinib) decreased mRNA levels of immunity-related genes (18, 19). FGFR1s are broadly expressed in crustacean tissues, with higher *Pc-FGFR4* mRNA levels in eyestalk ganglia, stomach, heart, intestine, and hepatopancreas and higher *Sp-FGFR3* levels in hepatopancreas, muscle, intestine, and heart (18, 19). Given their wide tissue expression, it is likely that FGFRs are involved in other processes. For example, in crayfish and other decapods, FGF controls blastemal growth during the initial stage of limb regeneration (79).

The PVRs were the most diverse of the four RTK classes. A total of 138 decapod PVR sequences were divided into two major subclades (PVR1 and PVR2; **Figures 1**, **11**, **Table 7**). PVR2 was further divided into PVR2A and PVR2B sequences, with PVR2B brachyuran-specific (**Figures 11C, D**, **Table 7**). The PVR tree was constructed by using PDGFR and VEGFR sequences jointly, as vertebrate VEGFR and PDGFR are not clearly differentiated in invertebrates (80). The evolution of VEGFRs and PDGFRs parallels the diversification and expansion of VEGFs in metazoans (81). An ancestral VEGFR/PDGFR ortholog, originally discovered in *Drosophila*, was designated PVR (PDGFR and VEGFR-Related Receptor), which diverged and led to PDGFR and VEGFR genes in vertebrates (80, 82). The lack of a clear distinction between PDGFR and VEGFR genes has contributed to inconsistencies in the annotation of homologous sequences in invertebrates. According to the classification proposed in **Table 7**, PVR1 sequences were identified in *G. lateralis*, *C. maenas* (4 isoforms), *C. borealis*, *E. sinensis*, *S. paramamosain*, and *P. leniusulus* (29). PVR2A sequences were identified in *G. lateralis* (2 isoforms), *C. maenas* (2 variants of one isoform), *L. vannamei* (2 isoforms; (70, 71), and *S. paramamosain*. PVR2B sequences were identified in *G. lateralis* (2 variants of one isoform), *C. maenas* (4 variants of one isoform), *C. borealis*, and *E. sinensis*.

PVR signaling is implicated in diverse physiological processes in decapods. In *L. vannamei*, five VEGFs and two VEGFRs are part of the immune response to viral infections; knockdown of VEGF and VEGFR expression reduces mortality, suggesting that PVR signaling supports viral replication (70, 71, 83–85). VEGF- and VEGFR-like immunoreactivities are localized in the eyestalk ganglia of the swamp ghost crab (*Ucides cordatus*), suggesting that VEGF is involved in neuron and glial cell differentiation and maintenance (86). In *S. paramamosain*, a VEGF-like gene (*Sp-vegfb*) has a role in lipid accumulation in the hepatopancreas and other tissues (30). In *P. leniusculus*, PVR signaling controls hematopoiesis by affecting extracellular transglutaminase (TGase) activity. Pl-PVR1 is expressed in hemocytes and hematopoietic tissue (HPT) (29). Sunitinib malate, a PVR inhibitor, decreases HPT progenitor cell migration and round cell morphology and increases HPT cell spreading and extracellular TGase activity (29).

Multiple sequence alignments of the catalytic domain aided the identification and classification of decapod RTKs. All four RTK classes shared three consensus motifs: the glycine-rich loop (GxGxFG), which plays a role in ATP binding; the aspartate-phenylalanine-glycine motif (DFG) near the activation loop; and the histidine-arginine-aspartate-leucine-alanine (HRDLA) motif in the catalytic loop (**Supplementary Material 3**) (87, 88). The only variation was in the PVR sequences, in which glycine replaced the arginine in the catalytic loop motif (HGDLA, **Figure 13**). Members of the EGFR class were readily identified by the high conservation in the catalytic domain; there were only four positions in the entire 305-amino acid sequence that differed (**Figure 7**). All the InsR had the same “VHRDLAARNC” sequence in the catalytic loop (**Table 3**; **Figure 4**), but the three InsR subtypes differed in sequences of a 20-amino acid motif located in the first FN3 domain in the N-terminal region (**Table 3**). The three FGFR subtypes differed in motif sequences (118 amino acids in FGFR1 and 117 amino acids in FGFR2 and FGFR3) that included the catalytic loop (**Table 6**, **Figure 10**). While conserved motifs are certainly useful in discriminating RTK types and subtypes, further work is required to elucidate their functional relevance. The complete conservation of the residues involved in ATP binding and peptide binding in EGFR1 (**Figure 7**) suggests that all members of the clade share the same catalytic properties. By contrast, the residues involved in ATP binding and peptide binding in the InsR, FGFR, and PVR sequences were not always conserved (**Figures 4**, **10**, **13**), suggesting that the subtypes within each clade differ in catalytic properties.

Processes such as development, growth, homeostasis, cell proliferation, and metabolism are regulated by growth factors, many of which are mediated by RTKs (3, 4, 82). In insects, RTK signaling controls molting by stimulating mechanistic target of rapamycin (mTOR)-dependent synthesis and secretion of molting hormones (ecdysteroids) by the prothoracic gland (82, 89–93). By contrast, the control of mTOR-dependent YO ecdysteroidogenesis by growth factor/RTK signaling has not been established (46). In *G. lateralis*, previous identification of *Gl-EGF*, *Gl-FGF*, *Gl-EGFR*, *Gl-FGFR*, and *Gl-InsR* in the YO transcriptome suggested that growth factors stimulate ecdysteroidogenesis, possibly through an autocrine mechanism (32, 33, 38, 46). The identification of multiple subtypes and isoforms provides a comprehensive catalog of RTK genes for functional analysis. Many of these RTKs were expressed in *G. lateralis* and *C. maenas* YO transcriptomes (**Tables 2**, **4**, **5**, **7**). The YO is primarily regulated by molt-inhibiting hormone (MIH), a neuropeptide that binds to a G protein-coupled receptor to inhibit ecdysteroid synthesis (41, 45, 46). A drop in MIH release from neurosecretory neurons in the eyestalk ganglia activates the YO and the animal enters early premolt (45). Growth factor receptors may sustain high rates of ecdysteroid synthesis by the committed YO during mid- and late premolt (46). For example, EGFR signaling in the prothoracic gland supports ecdysteroidogenesis during the lava to pupa transition in *Drosophila* (92).

## Conclusions

Bioinformatic and phylogenetic analysis using the CrusTome database yielded a rich diversity of hundreds of RTK contigs distributed across all crustacean taxa. The sequences were organized into InsR, EGFR, FGFR, and PVR clades, subclades, and isoforms, providing a framework for a classification nomenclature. Moreover, this extensive catalog of crustacean RTKs facilitates a systematic analysis of InsR, EGFR, FGFR, and PVR functions in various physiological processes, including, but not limited to, molting and growth, reproduction, regeneration, development and metamorphosis, nutrition and metabolism, and immunity, as well as their interactions with environmental stressors arising from climate change (94–96). Moreover, a greater understanding of growth factor/RTK signaling has important applications to sustainable aquacultural practices and the development of entirely new bioindustries, such as cellular agriculture and cultivated meats (28, 97–102).

## Data availability statement

The datasets presented in this study can be found in online repositories. The names of the repository/repositories and accession number(s) can be found in the article/**Supplementary Material**.

## Ethics statement

The manuscript presents research on animals that do not require ethical approval for their study.

## Author contributions

KF: Resources, Validation, Writing – review & editing, Writing – original draft, Visualization, Methodology, Investigation, Formal analysis, Data curation. JP-M: Writing – review & editing, Visualization, Validation, Supervision, Resources, Methodology, Investigation, Formal analysis, Data curation, Conceptualization. DD: Writing – review & editing, Resources, Project administration, Methodology, Investigation, Funding acquisition. DM: Writing – review & editing, Validation, Supervision, Project administration, Investigation, Funding acquisition, Formal analysis, Conceptualization.

## Supplementary material

Supplementary Material 1 Crustacean RTK sequences and classification.

Supplementary Material 2 Sequence alignments and phylogenetic tree figures of *G. lateralis* and *C. maenas* RTKs.

Supplementary Material 3 RTK sequence alignment files.

Supplementary Material 4 Decapod RTK motif multiple sequence alignment figures.
