# Supplementary material for: Phylogenetic and transcriptomic characterization of insulin and growth factor receptor tyrosine kinases in crustaceans
Source: Front Endocrinol (Lausanne). 2024 Apr 4;15:1379231. doi: 10.3389/fendo.2024.1379231 (PMC11024359; doi:10.3389/fendo.2024.1379231)

Supplementary Material #4. Decapod RTK motif multiple sequence alignment figures

Figure legends:

Supplementary Figure 1. Multiple sequence alignment and logo plot of the region proximal to the first FN3 domain across clades InsR1, InsR2, and InsR3. Includes Table 2 species (*Gecarcinus lateralis*, *Carcinus maenas, Cancer borealis, Sagmariasus verreauxi, Fenneropenaeus chinensis, Scylla paramamosain, Macrobrachium rosenbergii,* and *Eriocheir sinensis*). The alignment illustrates the composition and length of conserved regions within subclades that reflect putative differences in ligands and/or binding affinities between receptor types (in boxes). Conserved sequences diagnostic of RTKs are shown in bold. Partial sequences were excluded for ease of visualization and interpretation. MSA color scheme corresponds to similarities in physicochemical properties of amino acid residues.

Supplementary Figure 2. Multiple sequence alignment and logo plot of the catalytic region across clades FGFR1, FGFR2, and FGFR3. Includes Table 5 species (*Gecarcinus lateralis*, *Carcinus maenas*, *Procambarus clarkii, Litopenaeus vannamei, Eriocheir sinensis, Cancer borealis*, and *Scylla paramamosain*). The alignment illustrates the composition and length of conserved regions within subclades that reflect putative differences in ligands and/or binding affinities between receptor types (in boxes). Conserved sequences diagnostic of RTKs are shown in bold. Partial sequences were excluded for ease of visualization and interpretation. MSA color scheme corresponds to similarities in physicochemical properties of amino acid residues.

Supplementary Figure 3. Multiple sequence alignment and logo plot of the catalytic region across subclades PVR1 and PVR2. Includes representative Table 7 species (*Gecarcinus*

lateralis, *Carcinus maenas*, *Pacifastacus leniusculus, Cancer borealis, Eriocheir sinensis, Litopenaeus vannamei*, and *Scylla paramamosain*). The alignment illustrates the composition and length of conserved regions within subclades that reflect putative differences in ligands and/or binding affinities between receptor types (in boxes). Conserved sequences diagnostic of RTKs are shown in bold. Partial sequences were excluded for ease of visualization and interpretation. MSA color scheme corresponds to similarities in physicochemical properties of amino acid residues.

Supplementary Figure 1

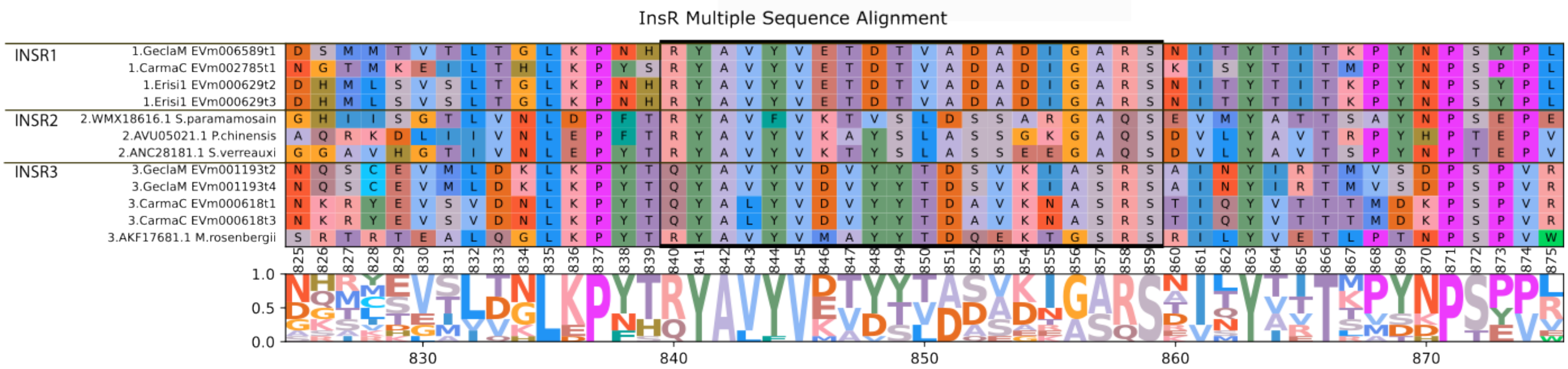

Supplementary Figure 2

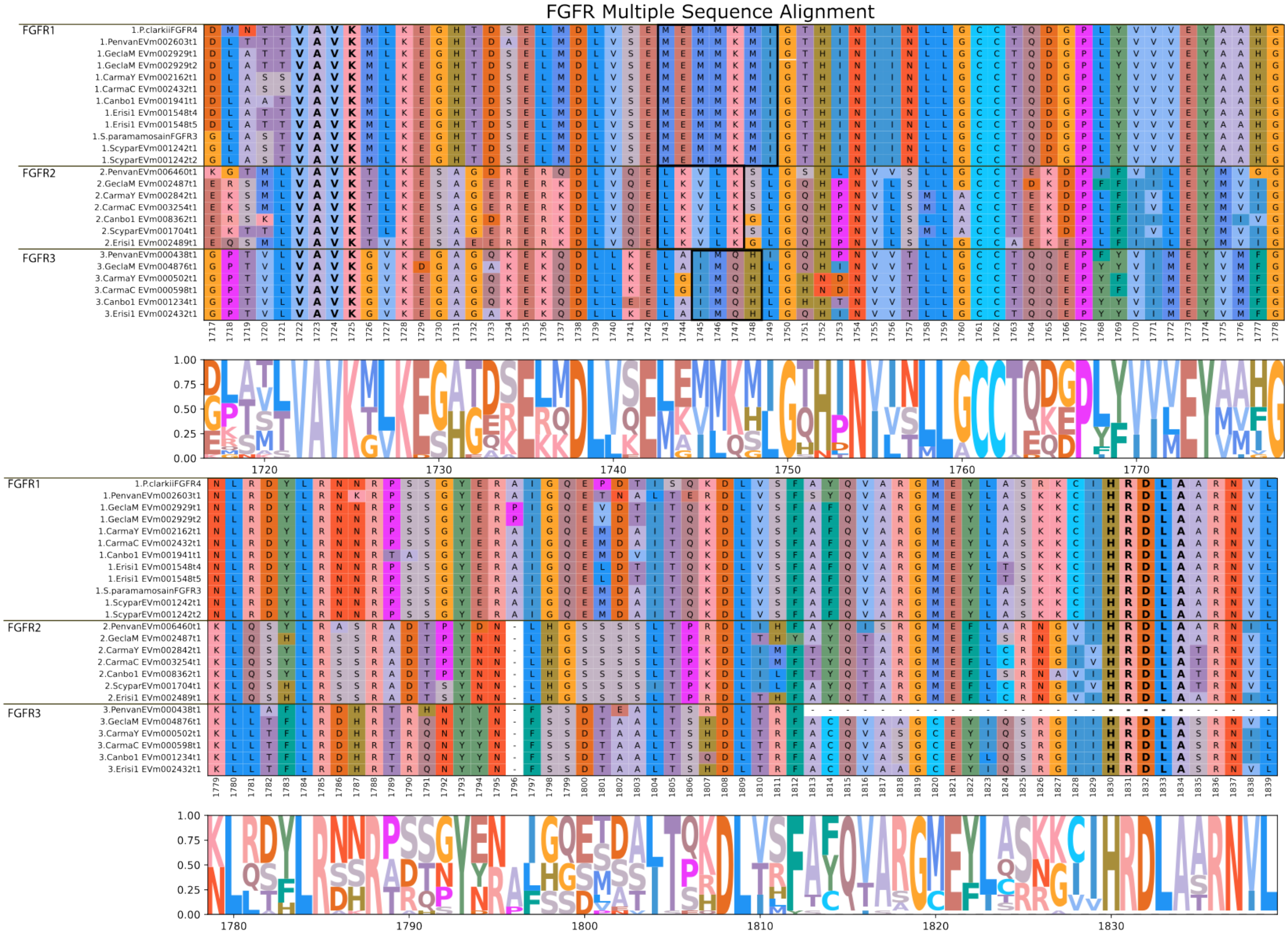

Supplementary Figure 3

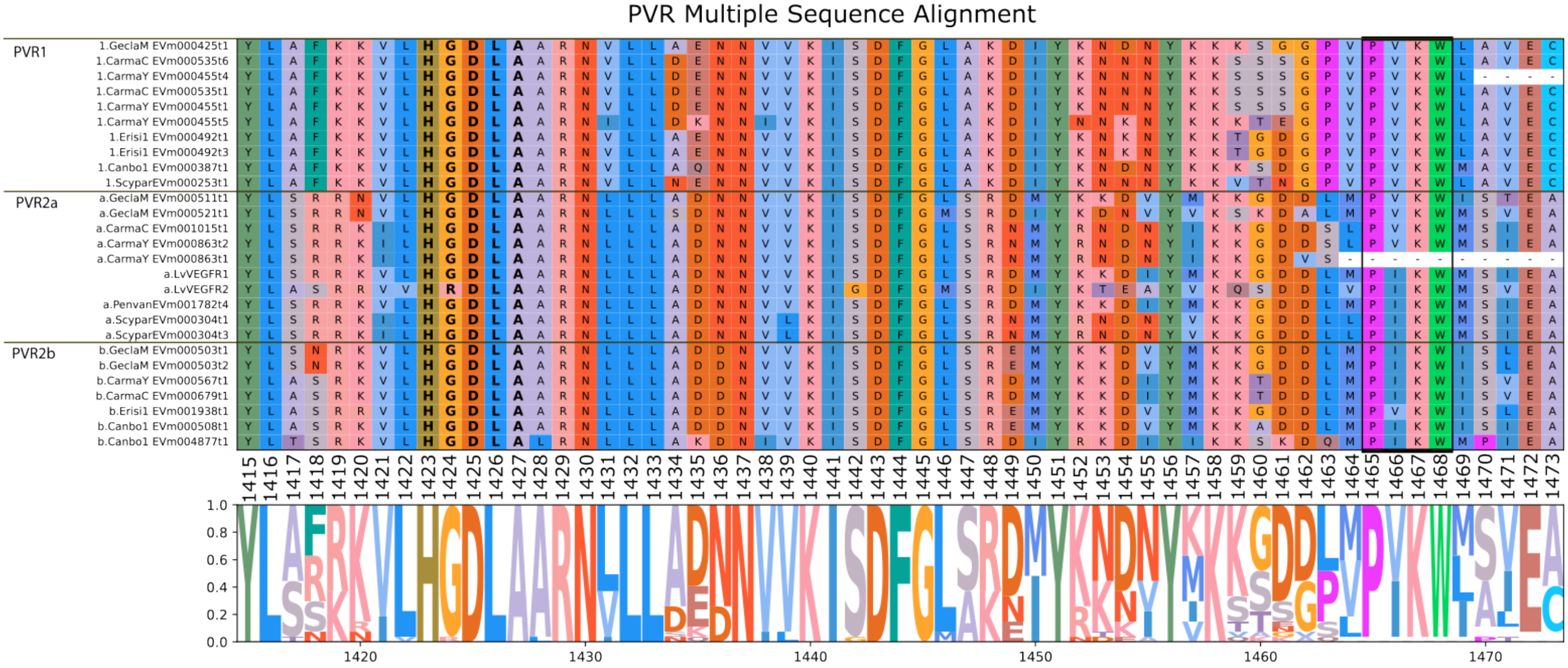

Supplement: Supplementary Material 1 — Crustacean RTK sequences and classification. [file DataSheet_1.zip › Supplementary material 4.pdf]
